# Multi-Omics in Kidney Disease Research: Technological Advances, Biomarker Discovery and Clinical Translation

**DOI:** 10.1017/erm.2026.10067

**Published:** 2026-08-11

**Authors:** Xuemei Gong, Xiaoxi Zeng, Ping Fu

**Affiliations:** 1 Department of Nephrology and Institute of Kidney Diseases, West China Hospital, Sichuan University, Chengdu, China; 2 West China Biomedical Big Data Center, West China Hospital, Sichuan University, Chengdu, China

**Keywords:** biomarker, kidney diseases, mechanism, multi-omics, precision medicine

## Abstract

**Background:**

Multi-omics research is reshaping precision nephrology by linking genetic susceptibility, epigenetic regulation, transcriptional state, protein abundance, metabolic activity and spatial tissue context within integrated analytical frameworks.

**Methods:**

We critically reviewed recent technological advances and applications of multi-omics in kidney diseases, with particular attention to biomarker discovery and clinical translation, and discussed the challenges and future directions of multi-omics integration and its application in precision medicine.

**Results:**

Emerging approaches, including single-cell and spatial omics, artificial intelligence-assisted analytics and integrative computational frameworks, have substantially improved the resolution of renal pathophysiology and molecular heterogeneity. These advancements not only reveal novel molecular pathways underlying chronic kidney disease, diabetic kidney disease, acute kidney injury and immune-mediated nephropathies, but also significantly facilitate biomarker discovery and therapeutic target prioritization. However, major challenges remain, including limited cohort diversity, high analytical complexity, lack of standardized pipelines, insufficient external validation and barriers to clinical implementation, such as cost, scalability and regulatory integration.

**Conclusions:**

Collectively, multi-omics strategies are reshaping kidney disease research and hold considerable promise for advancing precision diagnosis, risk stratification and personalized therapeutics. Future efforts should prioritize harmonized multicentre datasets, longitudinal validation, explainable artificial intelligence models and clinically actionable frameworks to bridge the gap between omics discovery and routine nephrology practice.

## Introduction

Kidney diseases impose a significant global health burden that severely impacts patients’ quality of life and lifespan, partly due to limited treatment options (Ref. 1). For instance, chronic kidney disease (CKD) affects approximately 10% of the global population; however, awareness remains below 10% (Refs 2, 3). Additionally, acute kidney injury (AKI) occurs in 7.0–18.3% of hospitalized patients without effective targeted therapies (Ref. 4).

These challenges reflect the intrinsic complexity and molecular heterogeneity of kidney diseases. Even within the same histopathological diagnosis, such as IgA nephropathy or diabetic kidney disease (DKD), disease progression and treatment response vary greatly. This heterogeneity arises from multilayered dysregulation across genetic, epigenetic, cellular and metabolic levels, which cannot be fully captured by conventional biomarkers like serum creatinine or albuminuria (Ref. 5). Moreover, renal biopsy is invasive and yields limited tissue, restricting comprehensive analysis (Ref. 6). Collectively, these limitations highlight the need for integrative approaches.

Recent advances in omics technologies have greatly improved the resolution and depth of biological investigation in kidney diseases (Ref. 7). However, single-omics approaches provide only fragmented insights and often fail to capture the complexity of heterogeneous disorders such as CKD and AKI. For example, genomics identifies inherited susceptibility but cannot reflect dynamic environmental influences (Ref. 8), whereas transcriptomics captures cellular activity without fully representing protein function or metabolic status (Ref. 9). Integrating multiple omics layers overcomes these limitations by linking genotype to phenotype through multidimensional molecular networks.

Consequently, multi-omics integration provides a comprehensive framework in which distinct molecular layers are integrated to elucidate disease pathogenesis. This systems-level framework enables improved characterization of disease heterogeneity, efficient use of limited biopsy material and resolution of cell-type-specific and spatially defined processes (Refs 7, 10, 11) (Figure 1). Recent developments in single-cell and spatial omics, together with computational pathology, have further enabled mapping of molecular interactions within intact tissue architecture (Ref. 12). Simultaneously, miniaturization and multimodal profiling now allow high-dimensional analysis from limited samples. These advances have accelerated mechanistic discovery, biomarker identification and disease classification, paving the way for precision therapeutics (Ref. 13). Within this framework, metabolomics serves as a vital functional readout by reflecting downstream biochemical activities and real-time pathophysiological alterations (Ref. 14). Unlike static genomic data, metabolomic profiles capture active cellular processes and environmental influences, providing direct insights into metabolic dysregulation and treatment responses. Recent bulk and spatial metabolomics studies have indeed unveiled profound disturbances in energy production, amino acid metabolism, lipid remodelling, purine pathways and uremic toxin accumulation across various nephropathies (Refs 13, 15).

**Figure 1.**
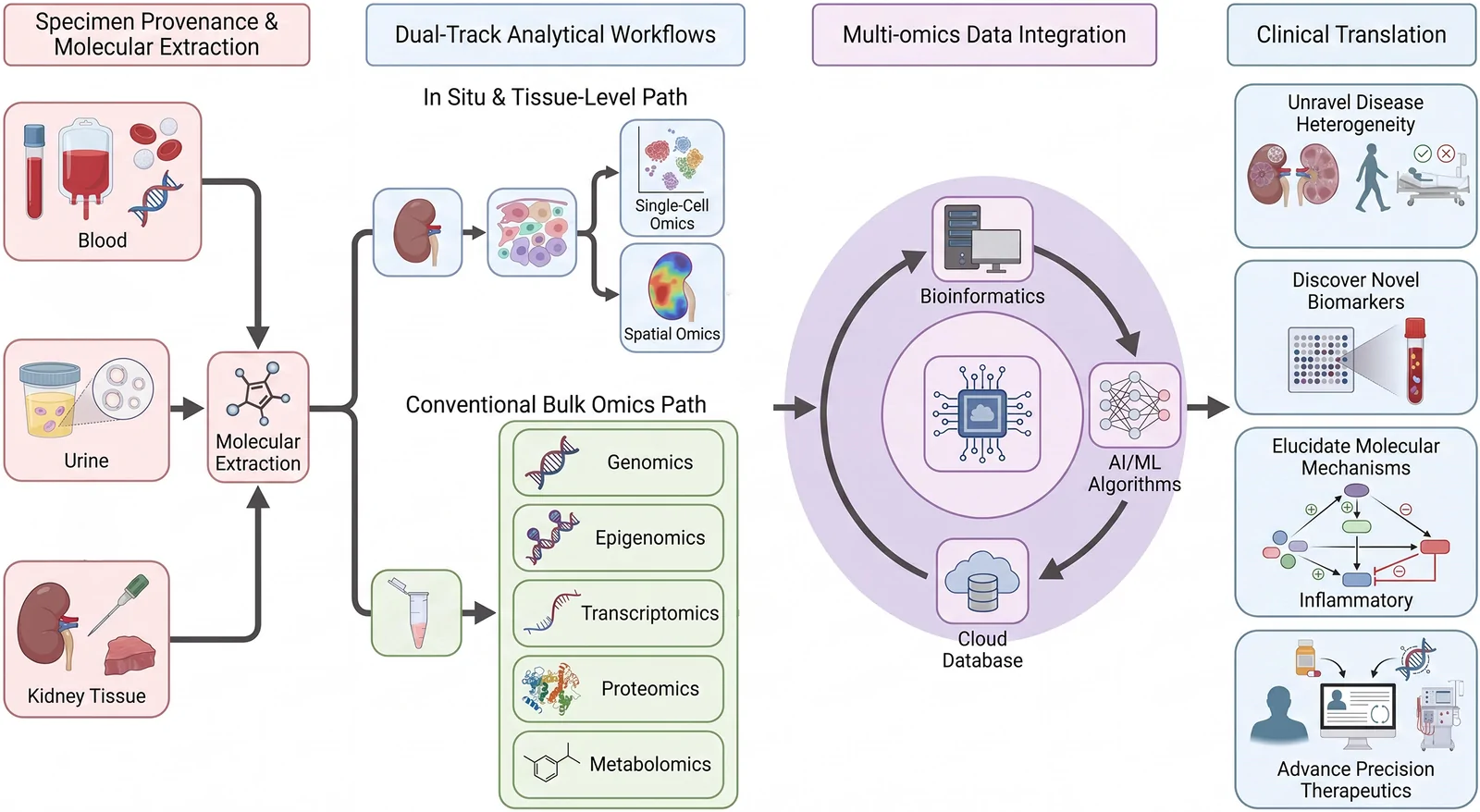
Multi-omics workflow in kidney disease research. *Note*: The research workflow transitions from specimen sources (blood, urine and kidney tissue) through dual-track analytical workflows. Track A (in situ and tissue-level analysis) utilizes single-cell and spatial omics to achieve cell-type resolution within a spatial context. Track B (conventional bulk omics) integrates genomics, epigenomics, transcriptomics, proteomics and metabolomics to provide deep molecular coverage. These parallel tracks converge into multi-omic data integration, facilitating clinical translation by unravelling disease heterogeneity, discovering novel biomarkers, elucidating molecular mechanisms and advancing precision therapeutics, ultimately aiming for precision medicine in nephrology. ctDNA: circulating tumour DNA; EVs: extracellular vesicles; scRNA-seq: single-cell RNA sequencing; snATAC-seq: single-nucleus assay for transposase-accessible chromatin using sequencing. Figure 1. long description.

Despite this progress, technical variability, lack of standardization, incomplete metabolite annotation and cross-platform validation challenges continue to hinder clinical application. As the field evolves quickly, a timely synthesis is vital to summarize recent advances and assess limitations. Therefore, this review provides an overview of multi-omics in kidney diseases, focusing on mechanisms, biomarkers and translational approaches into precision nephrology.

## Multi-omics technologies and integration strategies

### Core omics technologies: from molecular profiling to spatiotemporal resolution

#### Genomics and epigenomics

Genomics uses high-throughput sequencing techniques, including targeted next-generation sequencing (NGS) panels, whole-exome sequencing (WES) and whole-genome sequencing (WGS), to identify genetic variants associated with disease susceptibility and pathogenesis. Genome-wide association studies (GWAS) have further enabled the systematic identification of disease-associated loci, linking genotypes to phenotypes (Refs 16, 17). However, clinical translation remains limited, as most GWAS signals reside in noncoding regions with unclear regulatory functions, and individual variants exert modest effects. Polygenic risk scores (PRS) address this limitation by aggregating the effects of multiple variants, with genome-wide models improving predictive accuracy. PRS are now available for several kidney diseases, including CKD, DKD, IgA nephropathy and proteinuria (Ref. 18).

Epigenetics investigates how gene expression is regulated beyond the DNA sequence itself, encompassing mechanisms such as DNA methylation, histone modifications and noncoding RNA-mediated regulation (Ref. 19). These mechanisms are essential for regulating gene expression and may elucidate the elusive heritability observed in renal diseases. By mediating gene–environment interactions, epigenetic regulation substantially influences the progression of diverse kidney conditions, ultimately resulting in phenotypic modifications (Refs 20, 21). Aberrant epigenetic modifications have been implicated in AKI, CKD, DKD, glomerulonephritis and renal cell carcinoma, in part through modulation of metabolic pathways and transcriptional programs (Ref. 22).

#### Transcriptomics and single-cell omics

Transcriptomics profiles RNA expression, including both coding and non-coding RNAs, linking genomic variation to gene regulation through expression quantitative trait loci. In nephrology, transcriptomics has become a vital tool for understanding the molecular basis of different kidney diseases. By comparing gene expression profiles between healthy and diseased renal tissues, researchers can identify differentially expressed genes that are either upregulated or downregulated throughout disease progression (Ref. 23).

Advances in single-cell omics significantly deepen the understanding of kidney diseases. Since the advent of single-cell RNA sequencing (scRNA-seq) in 2009, single-cell methodologies have rapidly expanded to include genomics, epigenomics, proteomics and metabolomics. Single-cell transcriptomics, the most advanced method, has redefined kidney cell types and subtypes, resulting in comprehensive cell atlases and the discovery of specific markers. Both scRNA-seq and single-nucleus RNA sequencing (snRNA-seq) methodologies enhance understanding of renal cells in both healthy and diseased states (Ref. 24). Techniques such as single-nucleus assay for transposase-accessible chromatin using sequencing (snATAC-seq) strengthen single-cell epigenomics by enabling prediction of regulatory networks and transcription factor activity (Refs 25, 26). These advances provide a comprehensive view of dynamic cellular states and microenvironmental changes, particularly during disease progression.

#### Proteomics and metabolomics

As downstream functional layers, proteomics and metabolomics directly bridge static genetic predisposition and dynamic clinical phenotypes. Proteomics characterizes protein abundance, post-translational modifications and interaction networks, whereas metabolomics monitors small-molecule fluctuations reflecting active biochemical pathways. Because the kidney regulates filtration, tubular transport and metabolic homeostasis, circulating and urinary proteo-metabolomic signatures serve as sensitive indicators of both renal impairment and systemic dysregulation. Blood and urine are therefore invaluable, non-invasive matrices for biomarker discovery, disease stratification and longitudinal monitoring (Ref. 27). Clinically, anti-PLA2R autoantibodies in membranous nephropathy represent a landmark biomarker associated with disease activity and treatment response (Ref. 28). Additional antigens, such as THSD7A and amyloid A1, further refine prognostic assessment (Ref. 29). Metabolomics complements these insights by capturing dynamic metabolic alterations, with changes in amino acids, lipids and other metabolites that distinguish CKD patients from healthy individuals and predict outcomes (Refs 30, 31).

Both fields employ complementary untargeted and targeted strategies. Untargeted discovery relies on high-resolution mass spectrometry (MS) for unbiased, large-scale screening to chart novel pathways. Conversely, targeted strategies focus on predefined molecular panels, leveraging selective or parallel reaction monitoring (SRM/PRM) and isotope internal standards to achieve the high sensitivity and absolute quantification required for clinical assay development. Liquid chromatography-MS (LC–MS) remains the gold standard due to its superior sensitivity and broad chemical coverage. Gas chromatography-MS (GC–MS) provides resolution for volatile molecules, while nuclear magnetic resonance (NMR) offers exceptional reproducibility (Ref. 32). Recently, single-cell and spatial omics, alongside high-throughput platforms like Olink, SomaScan and MS imaging, have enabled the localized resolution of low-abundance molecules within specific renal tissue microenvironments (Refs 13, 33).

Nonetheless, analytical and biological bottlenecks hinder clinical translation. Data preprocessing, normalization and batch-effect correction remain non-standardized, limiting cross-study comparability (Ref. 33). This methodological challenge is compounded by confounding biological factors, as renal function trajectories, diet, medications and dialysis modalities substantially modulate proteo-metabolomic profiles. Furthermore, high-confidence metabolite annotation and protein modification mapping are often restricted by incomplete reference libraries and a scarcity of chemical standards. Overcoming these constraints requires rigorous quality control, standardized workflows and transparent data reporting (Ref. 34). Ultimately, integrating these functional layers with genomics, transcriptomics and experimental validation is essential for unravelling the multi-dimensional landscape of kidney diseases.

#### Spatial omics and multimodal integration

Spatial omics extends molecular profiling into tissue context by mapping genomic, transcriptomic and proteomic data within intact architecture (Ref. 35). This approach enables visualization of cell states, interactions and microenvironmental organization in three-dimensional space. Technologies such as spatial transcriptomics, multiplex fluorescence in situ hybridization and DNA-barcoded antibody labelling provide a more comprehensive understanding of molecular patterns in complex kidney tissues across multiple hierarchical dimensions (Ref. 36). When integrated with single-cell data, spatial omics enables precise cell-type annotation, inference of ligand–receptor interactions and identification of spatial niches. These approaches have been applied to characterize fibrotic microenvironments, spatial metabolic alterations in DKD and region-specific injury and repair programs (Refs 13, 37). Emerging technologies such as spatial CITE-seq now enable simultaneous co-mapping of transcriptomes and hundreds of protein epitopes at cellular resolution, while spatial ATAC-RNA-seq allows parallel profiling of chromatin accessibility and gene expression within the same tissue section (Ref. 38).

Collectively, multimodal integration provides a powerful framework for linking molecular alterations to tissue architecture, enabling anatomically resolved biomarker discovery and therapeutic targeting. As resolution approaches the single-cell and subcellular level, these technologies will be central to constructing comprehensive kidney atlases and advancing precision nephrology (Ref. 39).

## Data integration methodologies

The integration of multi-omics data is a critical step towards extracting meaningful biological insights. The choice of integration strategy is largely dictated by the research question, the nature of the available data and the hypothesis being tested. Integration methods can be broadly categorized into knowledge-based and data-driven approaches (Refs 40, 41) (Figure 2).

**Figure 2.**
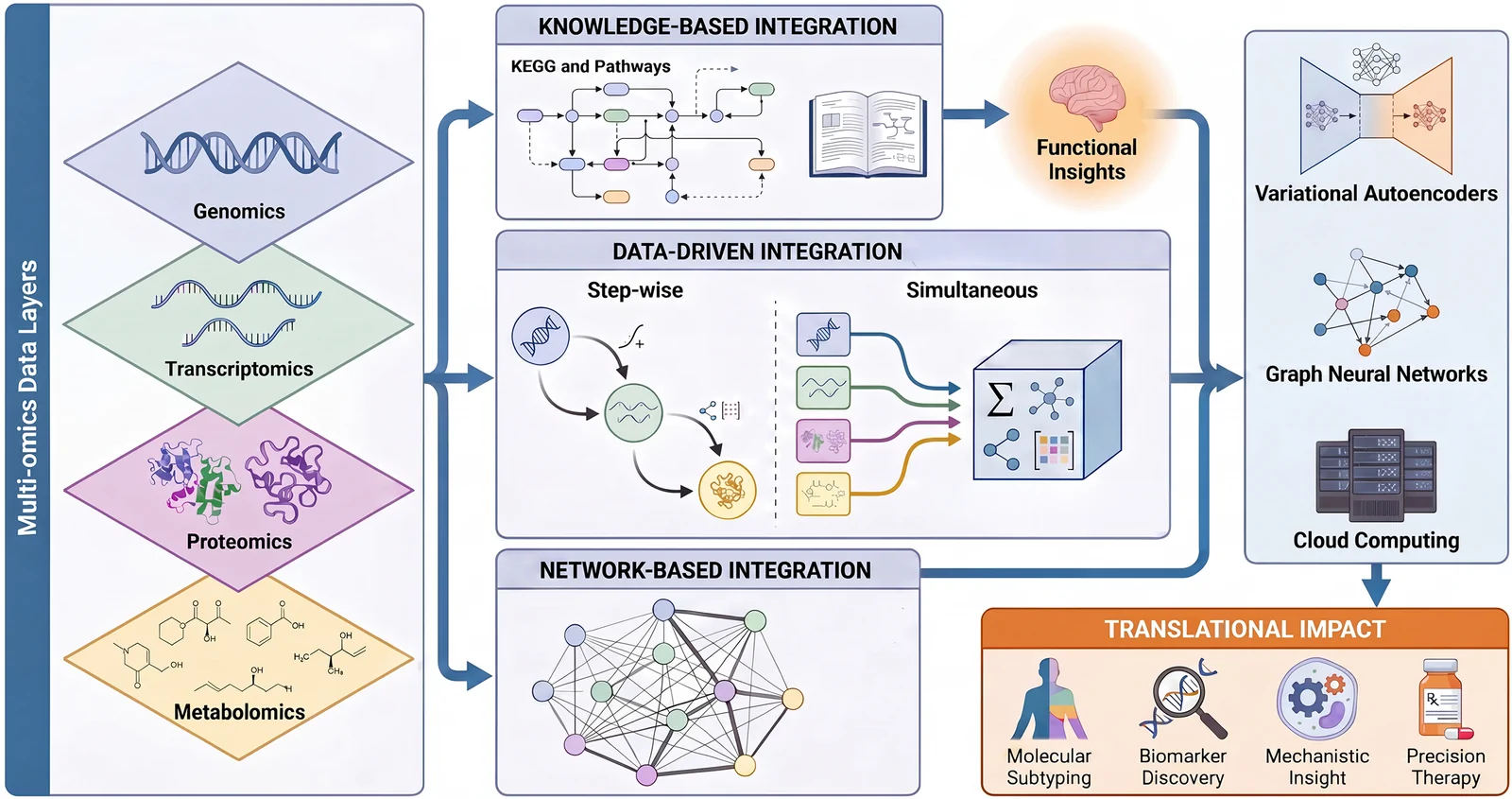
Computational strategies and methodologies for multi-omics data integration. *Note*: The workflow illustrates the transition from data input, comprising multi-layer omics, to diverse integration strategies. These strategies include knowledge-based integration (e.g., KEGG and biological pathways) to derive functional insights, data-driven integration utilizing stepwise or simultaneous approaches, such as joint latent space modelling and MOFA, and network-based integration via multi-layer networks. Advanced computational methods, including variational autoencoders, graph neural networks and cloud computing, facilitate these processes to achieve translational impact, specifically in molecular subtyping, biomarker discovery, mechanistic insight and precision therapy. MOFA, multi-omics factor analysis. Figure 2. long description.

### Knowledge-based integration

Knowledge-driven integration utilizes external information from databases (e.g., KEGG (Ref. 42), MetaCyc (Ref. 43) and Reactome (Ref. 44)) and scientific literature to establish connections among biological entities. This approach involves mapping multi-omics results onto prior knowledge frameworks, such as well-characterized metabolic or signalling pathways, to identify dysregulated processes across genomic, proteomic and metabolomic levels. A widely used technique is set-based enrichment analysis (Ref. 45), which evaluates whether a set of biologically relevant entities (e.g., differentially expressed or abundant molecules) is significantly enriched in specific functional annotation sets. By leveraging knowledge bases, this method assesses entities’ participation in common pathways, disease associations or co-localization patterns. The two principal variants are overrepresentation analysis (ORA) and functional set enrichment analysis (FSEA). ORA employs statistical measures, such as the hypergeometric test, to identify annotation terms that appear more frequently in a target list than would be expected by chance (Ref. 46). However, ORA considers only entities that surpass a predefined significance threshold, ignoring effect magnitudes and exhibiting sensitivity to the cutoff selection. In contrast, FSEA incorporates all measured entities and their quantitative values, enabling the detection of annotation sets characterized by either large changes in a few entities or subtle but consistent alterations across many (Ref. 47).

Nevertheless, knowledge-driven integration faces several constraints. These include reliance on the completeness and accuracy of existing annotations, inability to associate uncharacterized entities (e.g., novel metabolites or non-coding RNAs) and challenges in cross-platform identifier mapping due to synonymy and resolution disparities, particularly for lipids and metabolites. Additionally, ongoing curation of knowledge bases is necessary to prevent outdated or incomplete information, and these factors collectively limit the potential for novel discoveries.

### Data-driven integration

In contrast, data-driven integration employs statistical and machine learning techniques to infer relationships directly from the intrinsic structure of multi-omics data, without relying on prior knowledge. In studies focused on specific diseases or phenotypes, common applications include training predictors and classifiers and identifying multivariate biomarker candidates. Data-driven strategies are broadly categorized into stepwise integration, simultaneous integration and composite network approaches.

Stepwise integration sequentially analyses individual omics datasets and subsequently combines the results. It primarily includes ensemble integration and pairing association-based integration. Ensemble integration, such as *k*-nearest neighbours or Elastic Net, constructs independent predictive models for each omics dataset and integrates their outputs using methods such as majority voting or stacked generalization (Ref. 48). This approach enhances predictive performance, allows assessment of each dataset’s contribution and prevents datasets with numerous variables from dominating the analysis. However, it may overlook interdependencies between variables across different omics layers. Pairwise association-based strategies are often centred on genetic variation and quantitative trait loci (QTLs) identified through GWAS (Ref. 49). QTLs are genetic markers (e.g., single-nucleotide polymorphisms) that are significantly associated with the variation of quantitative molecular traits (e.g., the transcription level of a particular gene) (Ref. 50). By detecting overlapping QTLs across omics layers through colocalization analysis, this method systematically links genomic variation with other molecular phenotypes. It relies only on summary statistics, facilitating cross-study integration while circumventing data-sharing restrictions, and supports validation through independent replication. Although limited to pairwise associations, it is adaptable to various omics layers and offers a scalable framework for multi-omics analysis.

Simultaneous integration incorporates all omics data in a single modelling step to capture correlations within and across omics layers. It is subdivided into single-block and multi-block strategies. Single-block integration concatenates all omics data into a single matrix, enabling the direct application of conventional machine learning methods, such as clustering (e.g., K-means clustering), classification and regression (e.g., random forest, LASSO regression), or projection (partial least squares discriminant analysis (PLS-DA)). However, it ignores inherent heterogeneities among omics datasets, such as differences in scale, variance and number of features, which can introduce bias toward high-dimensional omics types. Multi-block integration explicitly accounts for the block structure of omics data (Ref. 51). Extensions of multivariate methods, such as O2-PLS (Ref. 52) for two omics layers and OnPLS (Ref. 53) for multiple layers, as well as frameworks like unsupervised regularized generalized canonical correlation analysis (Ref. 54) and supervised DIABLO (Ref. 55), allow balanced integration while revealing inter-omics associations. Nevertheless, simultaneous integration demands complete multi-omics profiles from the same samples, involves high computational complexity and is currently limited by the scarcity of large-scale datasets. Thus, although powerful, it requires careful selection of methods and thorough consideration of data heterogeneity.

Composite network approaches construct heterogeneous networks by integrating knowledge-driven and/or data-driven relationships to capture interactions across omics layers. The resulting network consists of nodes (biological entities, e.g., genes, proteins, metabolites) connected by edges that represent pairwise functional, biochemical or physical relationships (Ref. 56). Construction can be knowledge-driven, data-driven or hybrid. Hybrid approaches, which combine both annotation-based and empirically inferred interactions, support unbiased hypothesis generation and multi-omics exploration. For example, the HENA framework integrates GWAS data, protein–protein interactions and other molecular networks to identify Alzheimer’s disease-related genes using graph convolutional networks (Ref. 57). Once built, composite networks provide a reusable resource for identifying key nodes or predicting novel associations, independent of specific phenotypes. However, construction is challenged by identifier mapping, compound annotation, data format harmonization and weighting schemes. Analysis also demands sophisticated algorithms. Pre-built networks such as ConsensusPathDB (Ref. 58) and OmicsNet (Ref. 59) are increasingly valuable for facilitating such analyses.

Data-driven integration is powerful but not without caveats. It is highly susceptible to spurious correlations in high-dimensional data, necessitating rigorous validation and multiple testing correction. Results can be biologically challenging to interpret without functional context. Furthermore, these methods demand high-quality, preprocessed data and are sensitive to technical artifacts and batch effects.

### Emerging trends: AI-driven integration as a framework for multi-omics in kidney diseases

Driven by rapid technological advances and the accumulation of large-scale datasets, multi-omics integration is evolving rapidly. Artificial intelligence (AI), particularly deep learning, has become a key enabler, supporting non-linear integration across heterogeneous omics layers and translating complex molecular data into clinically actionable insights (Ref. 60). Architectures such as variational autoencoders (VAEs), graph neural networks (GNNs) and Transformers are especially effective for learning latent representations that capture both within-omics variation and cross-omics regulatory structure. VAEs provide a probabilistic framework for non-linear dimensionality reduction and are widely employed to address the inherent sparsity and noise in both single-cell and bulk multi-omics data. In single-cell integration, VAE-based approaches have become a dominant paradigm. For example, in spatial multi-omics, SpaOmicsVAE integrates VAEs with graph neural networks to model molecular profiles and spatial context, thereby identifying spatially coherent patterns. GNNs are suited for spatial multi-omics as cellular relationships can be naturally represented as graphs (Refs 61, 62). By encoding cells or locations as nodes and their interactions as edges, GNNs capture tissue architecture and cell–cell communication, aiding in identifying spatial domains and interaction networks. For example, CGIB employs graph convolutional networks for tumour classification, and graph attention networks are used in single-cell research for cell-type annotation and trajectory inference.

Recent studies have also introduced foundation models like MIDAA (Ref. 63) and OmicsAnalyst (Ref. 64), which integrate multiple modalities within a unified framework. Domain-specific models like Nephrobase Cell+ are optimized for kidney biology, outperforming general-purpose models like Geneformer, scGPT and UCE across the evaluated datasets. They produce concise, biologically coherent cell embeddings, balance batch-effect correction and cell-type preservation and perform well in cross-species zero-shot annotation tasks (Ref. 65).

Notwithstanding these advancements, significant challenges persist. Model interpretability is a major barrier, as opaque predictions hinder clinical trust and regulatory approval. Explainable AI techniques like SHAP and integrated gradients help address this problem but provide different levels of biological insight. Other hurdles include harmonization across platforms, batch effect correction and computational scalability, all crucial for integrating AI-driven multi-omics into clinical practice.

## Applications of multi-omics in kidney diseases

### A new paradigm for multi-omics in kidney disease research

Traditional kidney disease research has primarily relied on single-omics techniques applied to bulk renal tissue. While this has uncovered many disease-associated molecular changes, it faces three main limitations: it overlooks cellular heterogeneity in a highly complex organ comprising over 30 specialized cell types; it disrupts spatial context, obscuring region-specific interactions within structures such as the glomerulus and tubulointerstitium; and it captures only static snapshots, failing to resolve dynamic molecular changes during disease progression. As a result, many findings are correlational, complicating efforts to understand causation and develop targeted treatments. The advent of multi-omics technologies is gradually transforming this research landscape.

#### From single-dimension to multi-dimensional integration

Although single-omic technologies have identified numerous disease-associated pathways and candidate biomarkers, each captures only a limited layer of biological regulation. Multi-omics integration overcomes this limitation by combining complementary molecular information and enabling the reconstruction of multidimensional regulatory networks. By jointly analysing genomic, transcriptomic, proteomic, metabolomic and epigenomic data, researchers can uncover shared pathogenic mechanisms that may not be apparent from single-omics analyses alone.

Accumulating evidence from integrated multi-omics studies indicates that kidney disease progression is driven by several conserved biological programs despite substantial clinical heterogeneity. Among the most consistently identified mechanisms is mitochondrial dysfunction accompanied by impaired fatty acid oxidation (FAO) in proximal tubular epithelial cells (Refs 66, 67). Suppression of mitochondrial energy metabolism reduces ATP production, promotes oxidative stress and impairs adaptive tissue repair, thereby facilitating fibrosis and progressive loss of renal function (Ref. 68). In parallel, immune–metabolic reprogramming has emerged as another central feature, characterized by activation of inflammatory monocyte/macrophage populations, NF-κB/AP-1 signalling and extensive epithelial–immune cell communication (Ref. 69).

For instance, one study used multi-omics integration to systematically analyse immune-metabolic reprogramming in AKI induced by ischemia–reperfusion injury (Ref. 70). The results showed significant dysregulation of nucleotide metabolism in ischaemia-reperfusion injury (IRI), characterized by monocyte-specific activation. Using machine-learning analysis, the study identified Cmpk1 as a key hub gene, with its upregulation closely associated with mitochondrial dysfunction, pyroptosis and inflammatory responses. Spatial transcriptomics confirmed abundant Cmpk1 expression in renal tubulointerstitial monocytes, and in vivo and in vitro functional experiments also demonstrated that inhibiting Cmpk1 could significantly alleviate kidney injury and inflammation. This study revealed the central role of monocyte-driven nucleotide metabolism reprogramming in AKI and provided a potential therapeutic strategy targeting metabolic pathways.

Overall, multi-omics technologies are transforming kidney disease research from descriptive molecular profiling toward an integrated understanding of metabolic dysfunction, immune activation, cellular stress responses and fibrotic remodelling. Such systems-level insights are increasingly revealing common pathogenic networks that transcend individual disease etiologies and may provide a foundation for future precision nephrology.

#### From single-cell identification to spatial localization

Multi-omics has advanced from cataloguing differential gene expression to building detailed kidney cell atlases and understanding disease-specific cell states. Single-cell technologies allow detailed analysis of renal cell types and their regulatory networks. Recent studies identified major kidney cell populations, including previously unrecognized states and transitional phenotypes (Ref. 71). Notably, proximal tubular cells show high plasticity in response to injury, underscoring their role in disease progression (Ref. 72). These insights offer a basis for developing cell-specific therapies. Identifying injury-related and maladaptive tubular states suggests that interventions could target harmful cells while sparing repair mechanisms (Ref. 73).

However, dissociation-based single-cell methods lack spatial context, crucial for understanding cell interactions and injury spread. Spatial multi-omics technologies address this by mapping molecular profiles within tissue, revealing region-specific niches and signalling networks. Combined single-cell and spatial analyses have found inflammatory proximal tubular subpopulations in fibrotic areas in CKD, driven by HNF4α inactivation and AP-1/NF-κB activation, promoting disease via immune recruitment and fibroblast activation (Ref. 74). These insights inform therapies, such as targeting these signalling pathways, modulating epithelial–immune crosstalk or removing senescent, inflammatory cells to reduce fibrosis.

Ultimately, integrating these approaches shifts the focus from descriptive atlases to mechanism-based, microenvironment-targeted therapies, bridging molecular discovery with clinical application.

#### From correlation-based description to causal inference

Traditional omics research is limited by reverse causality and confounding bias, making it difficult to determine whether biomarkers are causes or effects of disease. Multi-omics approaches are expected to overcome these limitations. For instance, integrative analyses of genotype, transcriptome and metabolome data have enabled the construction of causal metabolic networks in CKD, identifying metabolites such as *N*-acetylisoleucine and decenedioic acid as potential drivers of disease via metabolic reprogramming. Mechanistic investigations suggest that these metabolites drive renal injury by modulating energy metabolism and oxidative stress via NAT8- and ACOT1/4-mediated metabolic reprogramming. Similarly, multi-omics studies have identified TNFRSF1A as a causal mediator of immune remodelling in diabetic nephropathy, supported by Mendelian randomization and experimental validation (Ref. 75). These approaches provide a stronger basis for therapeutic target prioritization and mark a shift toward mechanism-driven research.

In summary, the paradigm shifts described above are profoundly changing the research landscape in kidney diseases. Across studies, a more integrative picture is emerging: CKD and AKI are repeatedly associated with mitochondrial dysfunction, impaired fatty acid oxidation, purine and nucleotide metabolic remodelling, amino-acid and uremic-solute accumulation, immune-metabolic activation and extracellular-matrix remodelling. These convergent pathways are more informative than isolated biomarkers because they connect molecular alterations to tubular stress, inflammatory niches, fibrosis and clinical progression. A structured comparison of representative multi-omics studies is provided in Table 1. However, key challenges remain, including variability in sample quality, lack of standardization, limited longitudinal datasets and the need for robust cross-cohort validation. Addressing these issues will be essential to translate multi-omics insights into clinically actionable tools and advance precision nephrology.

**Table 1.** Representative multi-omics studies in kidney disease research Table 1. long description.

Study (year)	Disease type	Omics layers	Key findings	Clinical significance
Dumoulin et al. (2026) (Ref. 76)	DKD	Single-cell spatial Transcriptomics snRNA-seq	First high-resolution spatial atlas of DKD (500,000+ cells, 58 patients, 64 kidney samples), defining 11 reproducible “tissue niches”; Identified B-cell-enriched subgroup (B+ DKD) with highly organized tertiary lymphoid-like structures and active CXCL13-CXCR5 signalling, representing a rapidly progressing novel DKD subtype; Developed 14-protein plasma biomarker panel for non-invasive detection.	Enables precision patient selection for B-cell-targeted therapy; non-invasive biomarker panel improves risk prediction beyond traditional clinical models
Liu et al. (2025) (Ref. 77)	CKD	GWAS Bulk RNA-seq Single-nucleus multiome	Largest multi-ancestry GWAS for kidney function, identifying 1026 independent loci (97 previously unknown); Identified four kidney function-associated genomic loci specific to East Asian populations (BICC1, PKD2, ABCG2 and SIM1); Identified 1363 coding variants disrupting 782 genes; 601 genes are also targeted by both coding and regulatory variants.	Provides a comprehensive genetic blueprint of kidney function. The “Kidney Disease Genetic Scorecard” enables researchers and clinicians to pinpoint the specific genes and mutations most likely relevant to a given patient’s disease. Prioritizes a set of high-confidence, potentially causal genes for kidney disease, including *UMOD*, *SHROOM3* and *DAB2*, which are enriched with FDA-approved drug targets, thereby establishing a resource for genetics-based drug discovery.
Zhang, et al. (2025) (Ref. 70)	AKI	Bulk transcriptomics Metabolomics scRNA-seq Spatial transcriptomics	Multi-omics integration revealed nucleotide metabolism dysregulation in IRI; Single-cell analysis demonstrated monocyte-specific upregulation of nucleotide metabolism compared to other renal cell types; Multi-omics prioritized Cmpk1 as the central hub gene,Cmpk1 elevation correlated with monocyte pyroptosis, mitochondrial dysfunction and pro-inflammatory cytokine secretion.	Identifies Cmpk1 as a promising therapeutic target for AKI treatment. Provides a new perspective on the role of monocyte-specific metabolic reprogramming in kidney injury, enabling the development of targeted therapies for AKI.
Abedini, A. et al. (2024) (Ref. 78)	CKD	snRNA-seq; scRNA-seq; snATAC-seq; Spatial transcriptomics	Unbiased deconvolution of spatial data revealed four distinct microenvironments: glomerular, immune, tubule and fibrotic microenvironment (FME); Injured proximal tubule (iPT) cells are enriched within FMEs; two iPT subtypes were identified: iPT-VCAM1+ (metabolic function) and iPT-HAVCR1+ (cell adhesion/matrix-related). iPT-HAVCR1+ cells predominantly appear in CKD samples; Within FMEs, myofibroblasts (COL1A1+ SYNPO2+) show enrichment of focal adhesion and TGFβ signalling pathways; FME contains organized immune microenvironment with dendritic cells, plasma cells, B cells and T lymphocytes; matrix cells within FME express CXCL12, CCL19 and CCL21, signalling to immune cells.	FME spatial gene signature provides an improved prognostic tool compared to conventional histopathology, enabling better risk stratification of CKD patients. Comprehensive spatially resolved molecular roadmap of the human kidney and fibrotic process establishes a foundational resource for identifying therapeutic targets in kidney fibrosis. Reveals cellular interactions within FME (e.g., matrix-to-immune cell signalling) that may represent targetable pathways for anti-fibrotic therapy.
Lake et al. (2023) (Ref. 71)	Healthy reference & injured kidney (AKI/CKD)	snRNA-seq Spatial transcriptomics Three-dimensional imaging Epigenomics	Defined 51 main cell types and 28 injury-altered cellular states across nephron segments and interstitium; Molecular signatures predict maladaptive states associated with kidney function decline.	Comprehensive multimodal spatial cell atlas provides benchmark for cellular states, neighbourhoods and outcome-associated signatures
Li D et al. (2024) (Ref. 79)	DKD	Proteomics Single-cell RNA-seq	AKR1A1 identified as a molecular hub for cellular dysfunction in DKD; AKR1A1 insufficiency is associated with accumulation of succinate semialdehyde and altered glucuronate metabolism.	AKR1A1 and/or its metabolic end products serve as novel biomarkers for DKD risk prediction and early diagnosis
Li et al. (2024) (Ref. 20)	Kidney anatomy	Simultaneous single-cell ATAC/RNA sequencing Spatially resolved metabolomics	The same kidney cell type displays distinct transcriptomic, chromatin accessibility and metabolomic signatures depending on anatomic location; Nephron segments possess non-overlapping metabolic signatures; dysregulated lipid metabolism is observed in diseased PT cells; Integrated multimodal omics with clinical data identified PLEKHA1 as a disease marker; in vitro knockdown of PLEKHA1 increased gene expression in PT differentiation, suggesting possible pathogenic roles.	Provides the first large-scale spatially resolved multimodal single-cell atlas of human kidney anatomy; Highlights previously underrepresented cellular heterogeneity across kidney regions, enabling region-specific understanding of kidney physiology and disease; Identifies PLEKHA1 as a potential therapeutic target or disease marker linked to proximal tubule pathology.
Wang et al. (2025) (Ref. 80)	Lupus nephritis (LN)	Single-cell multiome Spatial transcriptomics Single-cell spatial transcriptomics GWAS	Identified VCAM1-expressing proximal tubule cells(PT_VCAM1) as part of an LN-specific inflammatory niche in the kidney cortex; PT_VCAM1 cells interact with myofibroblasts and immune cells to promote epithelial-mesenchymal transition (EMT); PT_VCAM1 cells originate from a failed-repair pathway, regulated by BACH2 transcriptional network.	Identifies PT_VCAM1 cells as a potential therapeutic target, linking genetic predisposition to cellular injury and disease progression in LN
Castro et al. (2025) (Ref. 81)	CKD	Transcriptomics Proteomics Metabolomics snRNA-seq	Discovered two molecularly distinct patient groups that, despite similar clinical profiles, showed significantly different event-free survival curves.	Enhances understanding of CKD pathophysiology; supports identification of new therapeutic targets and responder patient populations for precision medicine.
Si et al. (2024) (Ref. 82)	CKD	GWAS TWAS Proteomics	Identified 32 causal proteins for CKD via Mendelian randomization; 12 proteins replicated across CKD datasets and subtypes (FGF5, IDI2, INHBC, BTN3A2/3, UMOD, C4a/b, etc.); 20 novel proteins (GCKR, IGFBP–5, sRAGE, GNPTG, YOD1) also identified.	Identifies actionable drug targets for precision nephrology; proteins linked to multiple CKD subtypes suggest shared therapeutic mechanisms
Huang et al. (2025) (Ref. 83)	AKI	Proteomics Metabolomics	Identified 5 core metabolites (3-hydroxybutyrate, creatine, inosine, 3-hydroxymethylglutarate, myristic acid) as SA-AKI early signals; Proposed a diagnostic model named IC3 (inosine, creatine, 3-hydroxybutyrate) for early screening.	Non-invasive blood-based screening model for early SA-AKI diagnosis.

AKI, acute kidney injury; ATAC-seq, assay for transposase-accessible chromatin using sequencing; CKD, chronic kidney disease; DKD, diabetic kidney disease; GWAS, genome-wide association study; LN, lupus nephritis; scRNA-seq, single-cell RNA sequencing; snATAC-seq, single-nucleus ATAC sequencing; snRNA-seq, single-nucleus RNA sequencing; TWAS, transcriptome-wide association study.

### Research advances in multi-omics for CKD

#### Multi-omics enhances CKD mechanistic research

Integrative multi-omics approaches offer substantial advantages over single-omics or conventional methods by enabling the construction of dynamic, multidimensional biochemical interaction networks. These frameworks allow the tracking of temporal molecular interactions, thereby moving beyond static molecular connectivity maps.

#### Mitochondrial dysfunction and impaired fatty acid oxidation

Proximal tubular epithelial cells (PTECs) are highly energy-dependent and rely primarily on mitochondrial FAO for ATP production (Refs 67, 84). Across diverse CKD etiologies, including DKD, hypertensive nephropathy and glomerular disorders, multi-omics studies consistently identify impaired mitochondrial FAO as a central feature of disease progression. In CKD, persistent mitochondrial dysfunction drives oxidative stress, fibrosis and metabolic reprogramming, with epigenetic mechanisms regulating genes critical for mitochondrial homeostasis, such as *PMPCB* and *TFAM (85).*

Metabolic reprogramming and lipotoxicity are direct consequences of FAO failure. Metabolomic studies reveal accumulation of short- and middle-chain acylcarnitines and disrupted tricarboxylic acid (TCA) cycle intermediates, reflecting compromised mitochondrial energy metabolism (Refs 86, 87). Concurrently, defective FAO drives PTECs toward inefficient glycolysis, a shift mediated by HIF-1α and accompanied by upregulation of glycolytic enzymes such as HK2. This compensatory switch, however, occurs at the cost of ATP depletion, lactate accumulation and intracellular acidosis. Moreover, aberrantly elevated expression of fatty acid transporters CD36 and FATP2 promotes excessive free fatty acid uptake, while reduced ABCA1-mediated cholesterol efflux fosters lipid droplet accumulation and lipotoxicity (Refs 88, 89). The resulting lipotoxicity exacerbates mitochondrial dysfunction and oxidative stress, establishing a self-reinforcing pathogenic cycle. Activation of the FXR pathway has been shown to mitigate these effects by downregulating SREBP-1c, stearoyl-CoA desaturase-1 and acetyl-CoA carboxylase, while upregulating PPARα, CPT1a, PGC-1α, uncoupling protein-2 (UCP-2) and lipoprotein lipase (LPL), providing renal protection (Refs 88, 90).

Mitochondrial injury also links metabolic collapse to inflammation and fibrosis. Excess reactive oxygen species (ROS) trigger regulated cell death pathways, including necroptosis via RIPK1/RIPK3/MLKL, pyroptosis via NLRP3 inflammasome and Gasdermin D and ferroptosis via lipid peroxidation (Refs 84, 91). Severe damage leads to mitochondrial DNA (mtDNA) release, activating innate immune signalling through cGAS–STING and promoting pro-fibrotic cytokine production (Ref. 92).

In summary, integrative multi-omics evidence positions mitochondrial FAO failure as an upstream metabolic switch in CKD progression. By depleting energy supply, generating ROS and mtDNA signals and activating inflammatory cell death pathways, this defect transforms PTECs into central pro-inflammatory and pro-fibrotic effector cells, thereby linking metabolic injury to downstream pathological events.

#### Epigenetic memory and persistent pathological transcriptional programs

A defining feature of CKD is its continued progression despite effective control of initiating insults such as hyperglycaemia or hypertension. Recent evidence shows that epigenetic regulation plays a crucial role in creating and maintaining this “disease memory” (Ref. 93). ATP-dependent chromatin remodelling complexes have been linked to essential pathogenic processes such as fibroblast activation and inflammation (Ref. 94). Significantly, changes in chromatin state not only affect immediate transcriptional responses but also create long-lasting transcriptional memory, allowing disease-related genes to remain active (Refs 95, 96). Furthermore, chromatin remodelling interacts extensively with DNA methylation and histone modifications, forming a complex regulatory network that drives CKD progression (Ref. 97). These insights offer an epigenetic explanation for CKD’s irreversibility and point to potential therapeutic targets involving epigenetic modifications.

#### Extracellular matrix remodelling and fibrotic niches

Despite substantial etiological heterogeneity, progressive fibrosis remains the final common pathway leading to kidney failure. Recent integration of single-cell transcriptomics, spatial transcriptomics, proteomics and chromatin accessibility profiling has revealed that extracellular matrix (ECM) remodelling arises from coordinated interactions among injured tubular epithelial cells, activated fibroblasts and pericytes, endothelial cells and infiltrating immune populations (Refs 98, 99).

Among the pathways implicated, TGF-β signalling consistently emerges as the master regulator of fibrogenesis. Single-cell epigenomic and spatial proteomic analyses demonstrate that ECM stiffening promotes formation of YAP/TAZ–Smad3 complexes, which activate profibrotic enhancers and drive expression of collagen isoforms, fibronectin and α-smooth muscle actin (Ref. 100).

Notably, spatial atlases of human kidney diseases have identified four major tissue microenvironments – glomerular, tubular, immune and fibrotic compartments. Among these, fibrotic niches provide prognostic information beyond conventional histopathology and more accurately reflect disease progression. Integration of spatial transcriptomics with ligand–receptor interaction analyses further highlights extensive intercellular communication networks that coordinate inflammatory and fibrotic responses, offering new mechanistic insights and potential therapeutic targets in CKD (Ref. 78).

#### Multi-omics facilitates biomarker discovery in CKD

Ideal biomarkers should be non-invasive, easy to detect and reproducible. Currently, clinical biomarkers for kidney injury are limited. Traditional indicators like serum creatinine and proteinuria are not sensitive enough for early detection. Estimated glomerular filtration rate (eGFR) based on cystatin C is not affected by muscle mass or race, is more sensitive in detecting early decline in kidney function and is closely related to microvascular complications and long-term mortality (Refs 101, 102). However, it is more costly than creatinine and is easily affected by inflammation, making it less commonly used in clinical practice.

KIM-1 and NGAL are important markers for AKI. Currently, [TIMP2]*[IGFBP7] (NephroCheck) and NGAL have both been approved by the FDA for AKI (Ref. 103). In CKD, these markers also show increased levels in kidney gene expression and in urine (Ref. 104). For example, NGAL levels in serum and urine are related to eGFR and can independently predict CKD progression and cardiovascular complications (Ref. 105); KIM-1 is an important predictor of CKD progression and adverse renal outcomes. Studies have shown that high plasma KIM-1 levels are associated with a rapid decline in eGFR over 16 years and an increased risk of CKD (Ref. 106). Although many new biomarkers have emerged in recent years, the urinary peptidomics biomarker CKD273 remains one of the most extensively studied CKD biomarkers and has received FDA support (Ref. 107). The CKD273 classifier is a panel of 273 urinary peptides that enables early detection of CKD and prognosis of progression (Ref. 108). Multiple studies have shown that in individuals with normal eGFR, CKD273 can effectively assess and predict CKD progression, independent of confounding factors such as age and gender (Ref. 109). This conclusion has been validated in prospective clinical trials, demonstrating that CKD273 can identify patients at higher risk of progression with A2 proteinuria (Ref. 110).

Despite the excellent performance of these biomarkers in research, except for cystatin C, they have not yet been widely applied in clinical practice. The main barriers include the lack of standardized cutoff values, the absence of large-scale prospective validation and limited evidence of cost-effectiveness. Precise risk stratification is difficult to achieve using a single biomarker, but combinations of multiple markers or systems biology approaches are expected to provide more comprehensive and less biased risk identification methods. For example, AKR1A1 is a biomarker and regulator in DKD, with evidence across omics layers (Ref. 79). Transcriptomics shows decreased AKR1A1 in the renal tubular epithelial cells of DKD patients, confirmed at the protein level. Metabolomic profiling indicated that a deficiency in AKR1A1 leads to the buildup of succinic semialdehyde and glucuronic acid. GWAS identified a promoter variant (rs2229540) that reduces AKR1A1 transcription. Mechanistic evidence further supports a role for AKR1A1 in carcinogenesis, antioxidant activity and glucose metabolism, supporting its causal role in DKD. Though direct genotype-metabolite links need confirmation, findings collectively identify AKR1A1 as a biomarker and causal regulator in DKD.

Another representative example is endogenous adenine, which has emerged as both a predictive biomarker and a potential mediator of CKD progression. Metabolomic analysis of the CRIC cohort found that adenine is significantly correlated with progressive renal failure (Ref. 111). Spatial metabolomics further showed that adenine is present at low levels in healthy kidney tissue but is markedly enriched in DKD patients, particularly in glomerular matrix, atrophic tubules and sclerotic vessels, suggesting its potential involvement in fibrosis-related pathological processes (Ref. 112). Mechanistically, integrated multi-omics analyses indicate that abnormal accumulation of adenine mainly results from an imbalance in purine salvage and polyamine metabolism in proximal tubule cells, rather than simply increased synthesis. This metabolic reprogramming not only reflects cellular metabolic disorder but may also drive downstream pathological processes. Functionally, adenine is thought to promote ribosome biogenesis by activating the mTORC1–S6 kinase signalling pathway, thereby inducing cell hypertrophy and fibrosis (Ref. 113). In clinical translation, urinary adenine levels (or adenine/creatinine ratio) have been proposed as a non-invasive biomarker for predicting renal function decline and adverse outcomes, potentially independent of traditional indicators (Ref. 112).

Overall, adenine exemplifies a class of metabolic molecules with both mechanistic and biomarker potential. Its study highlights the critical role of multi-omics integration in connecting metabolic reprogramming, tissue microenvironment and disease progression, providing important directions for precise stratification and targeted intervention in CKD. Table 2 outlines the main functions, clinical uses and limitations of these biomarkers, highlighting their potential for integration into clinical practice.

**Table 2.** Potential biomarkers in kidney diseases – mechanisms, clinical significance, limitations and validation status Table 2. long description.

Biomarker	Disease	Mechanism	Clinical significance	Limitations	Validation status	References
CKD-273	CKD/DKD	A composite urinary peptide classifier based on 273 peptides, integrating signals from collagen degradation, inflammation and extracellular matrix remodelling.	Identifies high-risk individuals for CKD and DKD even when urinary albumin is normal; predicts cardiovascular events in CKD patients.	High cost; requires mass spectrometry platforms; clinical threshold and application scenarios need further standardization.	Received FDA letter of support but not yet fully approved for clinical use; lacks large-scale real-world data.	(109, 110, 114)
Cystatin C	AKI/CKD	Stably produced by all nucleated cells, freely filtered by the glomerulus and completely reabsorbed and catabolized in proximal tubules; more stable reflection of GFR than creatinine.	Better than creatinine for early AKI diagnosis; improves risk stratification and prognosis prediction.	Higher cost and limited availability in some settings; levels can be affected by thyroid disorders, corticosteroids, inflammation, obesity and other extra-renal factors.	In clinical use.	(115)
NGAL	AKI	A protein secreted by renal tubular epithelial cells upon tubular injury; detectable in both blood and urine.	Early AKI biomarker that can warn 24–48 hours before creatinine rise; in critically ill children, urinary NGAL ~125 ng/mL shows good performance for stage 2/3 AKI.	Lack of standardization; cost and accessibility issues; also elevated in systemic infection and inflammation.	FDA approved for clinical use.	(116)
KIM–1	AKI/CKD	Transmembrane glycoprotein highly expressed on proximal tubular epithelial cells after kidney injury and shed into urine; participates in apoptotic cell clearance and inflammation; sustained expression drives AKI-to-CKD transition.	Early warning and diagnosis of AKI; prediction of CKD progression.	Also significantly upregulated in RCC and other urological tumours.	In clinical validation phase.	(117)
suPAR	AKI/CKD	Soluble form of the urokinase-type plasminogen activator receptor; released upon inflammatory stimulation; involved in podocyte injury and disruption of the glomerular filtration barrier.	Predicts AKI onset and CKD progression; in IgA nephropathy, it independently forecasts the presence of segmental glomerulosclerosis (S) lesions and serves as a risk factor for poor prognosis.	Poor specificity; can be elevated by infection and systemic inflammation; no unified clinical threshold.	In clinical validation phase.	(118, 119)
FGF–23	CKD	A phosphate-regulating hormone secreted by osteocytes; rises early in CKD and contributes to mineral metabolism disorders and CKD-MBD.	One of the earliest markers of CKD-MBD, rising much earlier than abnormal serum phosphate; independently associated with CKD progression, increased CVD risk and ESKD mortality.	Levels influenced by multiple factors (phosphate intake, iron deficiency, inflammation, etc.) with biological variability; lacks standardized detection.	In clinical validation phase; anti-FGF–23 antibodies are in clinical development.	(120)
DKK3	CKD/AKI, Alport syndrome	Tubule-derived glycoprotein that is an antagonist of the Wnt signalling pathway; upregulated during kidney injury and fibrosis, detectable in urine.	Non-invasive marker of tubular injury and fibrosis; predicts CKD progression, cardiovascular events and all-cause mortality; significantly elevated early in paediatric Alport syndrome.	Affected by systemic inflammation and hypoxia; lacks standardization; ELISA cost is relatively high.	In clinical validation phase.	(121)
UMOD (Uromodulin)	CKD, ADTKD	Tamm-Horsfall protein specifically produced by epithelial cells of the thick ascending limb; serum levels reflect tubular mass and functional integrity.	Low urinary UMOD levels are associated with increased risk of AKI, CKD onset and rapid kidney function decline; associated with hypertension, ischemic stroke and metabolic syndrome; UMOD mutations cause ADTKD.	Urinary concentration is highly affected by hydration status, requiring normalization to urinary creatinine; serum and urine UMOD may reflect different pathophysiological aspects.	Widely recognized as a marker of tubular function; used in genetic diagnosis of ADTKD.	(122)
TIMP–2·IGFBP7	AKI	Urinary product of two cell cycle arrest proteins; reflects G1 arrest of tubular epithelial cells in response to stress.	First biomarker approved for AKI risk stratification (NephroCheck®); high specificity for moderate-to-severe AKI.	Primarily applicable to high-risk populations (ICU, major surgery); results may be influenced by certain drugs.	FDA approved; validated in multiple clinical trials.	(103, 123)
α1-Microglobulin	CKD/DKD	Low-molecular-weight protein freely filtered by the glomerulus and almost completely reabsorbed by proximal tubules; elevated urinary levels indicate impaired proximal tubular reabsorption.	Early marker of tubular injury; associated with diabetic kidney disease, CVD events and mortality.	Serum levels are affected by liver function; not specific for glomerular injury; lacks internationally standardized reference ranges and comparability between assays.	In clinical use; multiple studies have validated its utility as a tubular function marker.	(124)
L-FABP	AKI/CKD/DKD	Liver-type fatty acid-binding protein expressed in the cytoplasm of proximal tubular cells; released into urine upon oxidative stress or ischemia.	Early diagnostic and prognostic biomarker for AKI and CKD; independently predicts AKI risk stratification after cardiac surgery.	Not kidney-specific (also expressed in liver and intestine); false elevations can occur in systemic inflammation or liver disease; urinary levels are affected by proteinuria.	In clinical validation phase; uL-FABP is approved for clinical use in Japan.	(125, 126)
Urinary EGF	CKD/AKI/DKD	Epidermal growth factor expressed by distal tubules; promotes tubular cell proliferation and survival via autocrine/paracrine signalling; urinary levels reflect functional tubular mass.	Urinary EGF levels reflect functional tubular mass and repair status; low uEGF is associated with CKD risk, kidney function decline and increased cardiovascular events.	Primarily reflects distal nephron health, less sensitive for proximal tubular injury; requires standardized detection methods.	In clinical validation phase.	(127)
WFDC2	CKD/Fibrosis	A serine protease inhibitor upregulated in fibrotic kidneys of humans and mice; produced and released into blood by activated fibroblasts.	Potential biomarker and therapeutic target for renal fibrosis.	Most evidence from animal models; clinical translation data limited; extra-renal interference from its use as an ovarian cancer marker should be noted.	In clinical validation phase	(128, 129)
GDF15	AKI/CKD	Member of the TGF-β superfamily; widely expressed upon cellular stress (hypoxia, inflammation, oxidative stress); proximal tubular cells increase release during AKI.	Elevated circulating and urinary GDF15 levels are associated with increased CKD risk, faster kidney function decline and higher all-cause mortality.	Not kidney-specific (also elevated in cardiovascular disease, cancer, inflammatory diseases); lacks a kidney-specific threshold.	In clinical research phase.	(130, 131)
Urinary CK20	AKI → CKD	Cytokeratin 20; significantly upregulated in injured proximal tubular cells after AKI and shed into urine, detectable up to 7 days after injury.	In patients with acute decompensated heart failure and AKI, urinary CK20 effectively predicts the risk of AKI-to-CKD transition; early prediction of severe AKI and 30-day major adverse kidney events.	Mainly studied in specific clinical settings (cardiorenal syndrome, cardiac surgery); generalizability to other AKI aetiologies needs validation.	In clinical research phase.	(132, 133)
AKR1A1	DKD	Member of the aldo-keto reductase family; involved in detoxification of fatty acid metabolites; highly expressed in proximal tubular cells; deficiency leads to cellular dysfunction.	Novel molecular hub identified by multi-omics analysis in DKD; higher serum AKR1A1 levels in African American patients with type 2 diabetes are associated with slower eGFR decline, suggesting a protective role.	Primarily validated in African American populations; detection methods not standardized; limited validation in other ethnic groups.	In clinical research phase.	(79, 134)
ANGPT2	AKI and CKD	Angiopoietin-2, a marker of endothelial activation; competes with angiopoietin-1 for Tie2 receptor binding, promoting vascular instability and inflammation.	Elevated circulating ANGPT2 levels correlate with disease severity in sepsis-associated AKI; in CKD, ANGPT2 is associated with albuminuria, kidney function decline, renal outcomes and cardiovascular complications.	Not kidney-specific; also elevated in various cardiovascular and inflammatory diseases.	In clinical research phase.	(135)

*Note*: The table summarizes 15 potential biomarkers for kidney disease, with each row listing the associated disease, mechanism, clinical significance, limitations, validation status and references.

ADTKD, Autosomal Dominant Tubulointerstitial Kidney Disease.

### Acute kidney injury

#### The role of multi-omics in advancing AKI mechanistic studies

*Heterogeneous injury phenotypes of proximal tubular cells*. PTECs are the most vulnerable cell type in AKI, relying heavily on fatty acid oxidation, containing many mitochondria and being highly susceptible to ischemia, nephrotoxins and inflammation (Ref. 136). However, not all PTECs respond the same way to injury. Single-cell studies have shown that PTECs display a range of injury types after AKI. An integrated multi-omics analysis of human transcriptomic data identified three key genes: EGF, VCAM1 and SUGCT. Among these, SUGCT expression was associated with immune cell infiltration and mitochondrial dysfunction and was specifically enriched in PTECs (Ref. 136). This connects mitochondrial metabolic issues with changes in the immune environment, suggesting PTECs are not just passive victims but active players in immune modulation.

In sepsis-related AKI, a combined bulk RNA-seq and scRNA-seq analysis identified Hmox1 as a marker of PTEC injury. Its increased expression in specific PTEC subpopulations supports its potential as a diagnostic marker (Ref. 137). Single-cell analysis has also shown high levels of PECR and TP53I3 in PTECs and loop of Henle cells, along with associations with lactylation-related pathways, indicating that metabolic changes may have previously unrecognized roles in AKI development (Ref. 138).

*Fine-scale resolution of immune cell subsets.* AKI pathogenesis and progression heavily rely on immune system activity, but immune cell diversity has long been difficult to characterize due to technical limitations. Single-cell technologies are revolutionizing this area. A recent study combined scRNA-seq datasets from five etiological models across multiple time points to create the first single-cell atlas of neutrophils in AKI. It identified nine distinct subsets – six of which were new to AKI and three not previously reported in the literature (Ref. 139). These subsets showed significant functional differences in phagocytosis, antigen presentation, inflammatory control, vascular regulation and fibrosis. Spatial neighbourhood analysis revealed a neutrophil-centred microenvironment characterized by specific interactions between neutrophils and the thick ascending limb of the loop of Henle, thereby uncovering a new form of intercellular communication.

Zhang et al. combined scRNA-seq and spatial transcriptomics to map the dynamic and spatial distribution of renal macrophages in an AKI-to-CKD progression model (Ref. 140). They identified functional differences between resident and monocyte-derived macrophages and discovered a new extracellular matrix-remodelling macrophage subset that interacts with fibroblasts through IGF signalling pathways. Single-cell omics have significantly transformed our understanding of macrophage roles in AKI, highlighting new therapeutic targets to prevent AKI and its chronic progression.

*Molecular mechanisms of AKI-to-CKD transition.* Post-AKI renal repair is highly heterogeneous: some patients achieve complete recovery, whereas others develop maladaptive repair characterized by inflammation, fibrosis and progressive decline in kidney function leading to CKD. Understanding the molecular factors behind these pathways is crucial to preventing the AKI-to-CKD transition.

A landmark study integrating lineage tracing with single-nucleus multi-omics provides key insights into this process. By labelling early-proliferating cells following AKI with Ki67 and performing joint snRNA-seq and snATAC-seq on the same nuclei, the authors generated a high-resolution dataset comprising over 80,000 nuclei (Ref. 141). This analysis revealed that AKI induces a widespread cell cycle response across both epithelial and non-epithelial compartments; however, the subsequent repair trajectories diverge markedly. Notably, a subset of pro-inflammatory tubular cells persists for months, indicating ongoing pathology. Differences in gene regulation and transcription factors distinguish adaptive from maladaptive repair, with maladaptive cells showing sustained inflammatory signals.

Importantly, these findings indicate that even morphologically recovered kidneys retain persistent transcriptional and epigenetic alterations, which may predispose to chronic disease. This supports early intervention to reprogram maladaptive cell states before fibrosis becomes irreversible. Epigenetic regulation further contributes to this transition. DNA methylation analyses reveal key genes, including Atp1a3, Ncf1, Lpl and Slc27a2, associated with renal outcomes, with pathway enrichment analyses highlighting PPAR signalling and metabolic regulation (Ref. 142). Pharmacological inhibition of DNA methyltransferases reduces fibrosis and restores lipid metabolism in models, showing that epigenetic changes are modifiable drivers of disease (Ref. 142). Complementing these observations, cross-species multimodal atlas studies have further demonstrated that distinct injury-related cell states are organized within specific niches and shift during disease. Gene networks identify critical transcription factors (e.g., SOX4, SOX9, NFKB1) that drive these transitions, linking them to outcomes (Ref. 143). These approaches also uncover biomarkers predictive of progression, aiding risk assessment and treatment timing.

Overall, sustained, spatially organized transcriptional and epigenetic reprogramming underpins the AKI-to-CKD transition. Multi-omics enhances understanding, identifies mechanisms and highlights opportunities for intervention.

### Biomarker discovery in AKI driven by multi-omics technologies

AKI diagnosis primarily depends on serum creatinine and urine output, which are not sensitive indicators of early injury. Recent research emphasizes biomarkers indicative of subclinical renal damage, including those for tubular injury (NGAL, NAG), glomerular filtration (cystatin C), cell-cycle arrest (IGFBP-7, TIMP-2), inflammation (interleukins, CCL14) and metabolic stress (lactate) (Refs 144, 145). However, only a few have progressed from observational evaluation to clinical practice.

The combination of cell-cycle arrest markers IGFBP-7 and TIMP-2 predicts moderate-to-severe AKI in critically ill patients, outperforming serum creatinine (Ref. 123). NGAL is a key biomarker for kidney injury, released from renal tubular cells, neutrophils and other epithelial cells in response to tissue damage. It has demonstrated utility in both AKI and CKD, offering advantages over serum creatinine due to its early response to renal insult (Ref. 105).

Recent research has focused on biomarkers associated with severe AKI phenotypes, such as CCL14. Meta-analyses indicate that CCL14 outperforms other markers in predicting persistent AKI with high accuracy across ICU, sepsis and postoperative groups (Ref. 146). It also correlates with outcomes such as oliguria and the need for kidney replacement therapy (Ref. 147). However, cutoff values for CCL14 are mainly derived from a single study and lack extensive validation across diverse populations. To date, no clinical trials have confirmed that CCL14-guided strategies improve patient outcomes.

Advances in high-throughput technologies have driven biomarker research towards integrated multi-omics approaches, revealing common pathways in AKI, including immune dysregulation, cellular stress responses and metabolic reprogramming. For example, a metabolomics study of sepsis-related AKI identified urinary 3-methylhistidine as a potential biomarker, with a diagnostic AUC of 0.86 in a clinical cohort, rising to 0.89 when combined with clinical data (Ref. 148).

Advances in single-cell techniques and extracellular vesicle research have enhanced biomarker discovery. Urinary extracellular vesicle surface CD35 showed an AUC of 0.89 for SA-AKI detection and 0.84 for subclinical AKI, and correlated with severity, persistence, mortality risk and progression (Ref. 149). Multi-omics analyses suggest that CD35 primarily originates from injured podocytes, indicating its potential as an early marker of injury. Similarly, combining renal transcriptomics, WGCNA and clinical data identified SUGCT as a key AKI marker; low SUGCT expression was associated with impaired mitochondrial function and heightened immune activity, and spatial transcriptomics confirmed its presence in regions involved in oxidative phosphorylation (Ref. 136). Despite progress, most emerging biomarkers remain in the early stages of discovery and have not yet reached clinical validation (see Table 2). To be clinically impactful, biomarkers need to go beyond diagnostic accuracy and provide actionable information, such as distinguishing AKI subtypes, enabling early risk stratification, guiding therapy and monitoring response, while remaining practical for routine use.

## Challenges in applying multi-omics to kidney disease research

### Limitations in sample acquisition and biobanking

Limited availability of high-quality biological specimens hampers kidney multi-omics research. Kidney diseases exhibit high molecular diversity among patients, and their progression complicates longitudinal sampling (Ref. 150). Renal biopsy is an important source of disease-relevant kidney tissue, but its invasiveness and associated risks make it unsuitable for large studies. Most studies therefore rely on surrogate samples like blood and urine, which only partially reflect renal pathology. Multi-omics studies also lack standardized workflows, quality control and data protocols. For instance, variations across platforms, reagents and bioinformatics pipelines lead to batch effects that hinder pooled analysis and validation (Ref. 151).

To address sample scarcity and biological heterogeneity, international consortia such as the Nephrotic Syndrome Study Network (NEPTUNE), the European Renal cDNA Bank (ERCB), the EURenOmics Consortium and the Kidney Precision Medicine Project (KPMP) have established disease-specific biobanks to enhance statistical power and research reproducibility. However, complete standardization of sample handling remains difficult. For example, KPMP provides Standard Operating Procedures (SOPs) for renal biopsy tissue dissociation, but variability in tissue dissociation and cell viability affects data quality and comparison. Differences in data formats and metadata standards also impede cross-repository analysis (Ref. 152). To address these limitations, the field should adopt unified data structures and metadata standards spanning sample processing, data generation and reporting.

### Technical barriers to data integration

Cross-modal data integration continues to be a major technical challenge. The main obstacle involves data heterogeneity and mismatched scales (Ref. 153). Different platforms produce data with diverse feature sizes, distributions and ranges. Correlations between omics layers tend to be weak or moderate due to biological and technical factors. This heterogeneity makes simple concatenation biased and ineffective. Data standardization and harmonization are therefore crucial for multimodal integration. Moreover, model training also faces the classic “curse of dimensionality,” making it vital to reduce dimensions and select the most influential features and variables. To address these challenges, new machine learning approaches have been developed, such as novel neural fuzzy systems that combine rule-based layers, deep structures and fuzzy layers for classifying kidney cancer subtypes (Ref. 154), and algorithms like RECODE that reduce noise in scRNA-seq data (Ref. 155).

### Barriers to clinical translation: From biomarker discovery to clinical application

Although multi-omics technologies have expanded the molecular understanding of kidney diseases, their clinical use faces systemic barriers. The main challenge is not discovery but the integration and usability of evidence.

First, there is a mismatch between validation frameworks and the biology of kidney diseases. The kidney’s functional reserve makes traditional endpoints, such as serum creatinine, insensitive in the early stages. Multi-omics biomarkers must demonstrate predictive value during this ‘subclinical stage’ but lack standard reference points, relying instead on surrogate endpoints or long-term outcomes, thereby prolonging translation and adding uncertainty (Ref. 156). Current systems are not yet adapted to molecular medicine.

Second, evidence extrapolation is limited, affecting biomarker reliability. Most studies are small, single-centre and conducted at a single time point, ignoring time dependence and molecular changes of kidney diseases. This causes instability in real-world settings, highlighting the need for “dynamic trajectory modelling” instead of “static discrimination.”

Third, interpretability and standardization remain major obstacles. Most multi-omics biomarkers are high-dimensional signatures with limited biological interpretability and no standardized detection methods, making clinical translation difficult. They also require interdisciplinary expertise, often lacking in clinics. Although newer guidelines promote standardization, no clinical pathways have been established to integrate these results into decision-making (Refs 11, 157).

Finally, economic and risk–benefit issues are key. Sensitive detection can expand “disease boundaries,” risking overdiagnosis, overtreatment, increased costs and patient burden. The value of biomarkers should be evaluated within broader frameworks that consider cost-effectiveness, risk stratification and their impact on clinical decision-making (Ref. 158).

In summary, the key pathway for clinical translation of multi-omics involves a “discovery to decision” loop. This means creating dynamic validation frameworks using longitudinal data, standardizing detection methods, developing interpretable models and assessing clinical and economic impacts through real-world studies. For metabolomics, this loop should additionally include transparent annotation confidence, targeted assay confirmation, reference-material use where feasible and cross-laboratory ring trials. Only by bridging technological feasibility and clinical usability can precision nephrology truly advance.

## Future application prospects of multi-omics in kidney diseases

### Emerging omics technologies

Recent advances in omics technologies are shifting kidney disease research from static molecular profiling toward dynamic, spatially resolved characterization of disease processes. Rather than merely cataloguing molecular alterations, emerging approaches seek to define how cellular interactions, metabolic programs and regulatory networks evolve during disease progression.

Among these developments, spatially resolved omics has transformed the investigation of tissue heterogeneity (Ref. 12). Spatial transcriptomics, proteomics and metabolomics preserve tissue architecture while simultaneously profiling molecular features, enabling direct interrogation of cellular communication within glomerular, tubular, vascular and interstitial compartments. In kidney diseases, spatial multi-omics has uncovered region-specific metabolic perturbations, inflammatory niches and fibrotic microenvironments that are largely inaccessible to conventional bulk analyses (Refs 99, 159). For example, spatial metabolomics has identified focal adenine accumulation within fibrotic lesions and compartment-specific glycan remodelling in DKD, highlighting the importance of spatial context in disease pathogenesis and therapeutic targeting (Ref. 13).

Parallel advances in single-cell multi-omics now permit simultaneous interrogation of multiple molecular layers, including the genome, epigenome, transcriptome, proteome and metabolome, within individual cells (Ref. 39). These approaches facilitate reconstruction of cell-specific regulatory circuits and identification of rare pathogenic populations that may disproportionately drive disease progression (Refs 9, 160). Integration of single-cell transcriptomics with emerging single-cell metabolomics further provides a framework for linking transcriptional states to functional metabolic programs, thereby offering a more mechanistic view of renal pathophysiology.

Attention is also shifting toward technologies that capture biological dynamics. Metabolic flux analysis, isotope tracing and high-resolution metabolic imaging enable quantification of pathway activity in vivo, overcoming the static nature of conventional metabolomics (Refs 161, 162). By distinguishing active metabolic flux from metabolite accumulation, these approaches provide functional insight into metabolic reprogramming during kidney injury and repair (Ref. 163).

Artificial intelligence is expected to become an integral component of this framework. Large-scale foundation models, including scGPT and kidney-specific platforms such as Nephrobase Cell+, are increasingly being applied to multimodal data integration, cell-state prediction, perturbation modelling and cross-omics inference. Such models may accelerate the transition from descriptive molecular profiling to predictive and mechanistic modelling of disease trajectories.

Despite these advances, several barriers remain. Standardized protocols for sample processing, data generation and computational analysis are needed to improve reproducibility across studies. Integrating heterogeneous datasets generated across platforms and populations remains challenging, while longitudinal multi-omics cohorts are still scarce. Furthermore, translating molecular signatures into clinically actionable biomarkers and therapeutic targets will require rigorous validation in large, well-characterized patient populations.

Collectively, the convergence of spatial multi-omics, single-cell technologies, fluxomics and artificial intelligence is redefining kidney disease research. Future efforts will increasingly focus on integrating spatial, temporal and functional dimensions of biological information, advancing nephrology from molecular description toward predictive, systems-level precision medicine.

### Multimodal clinical data integration

Multimodal clinical data integration is a vital part of precision nephrology. By combining omics layers with medical imaging, histopathology and electronic health records (EHRs), researchers can build detailed molecular maps of kidney diseases, improving disease classification and risk prediction (Figure 3).

**Figure 3.**
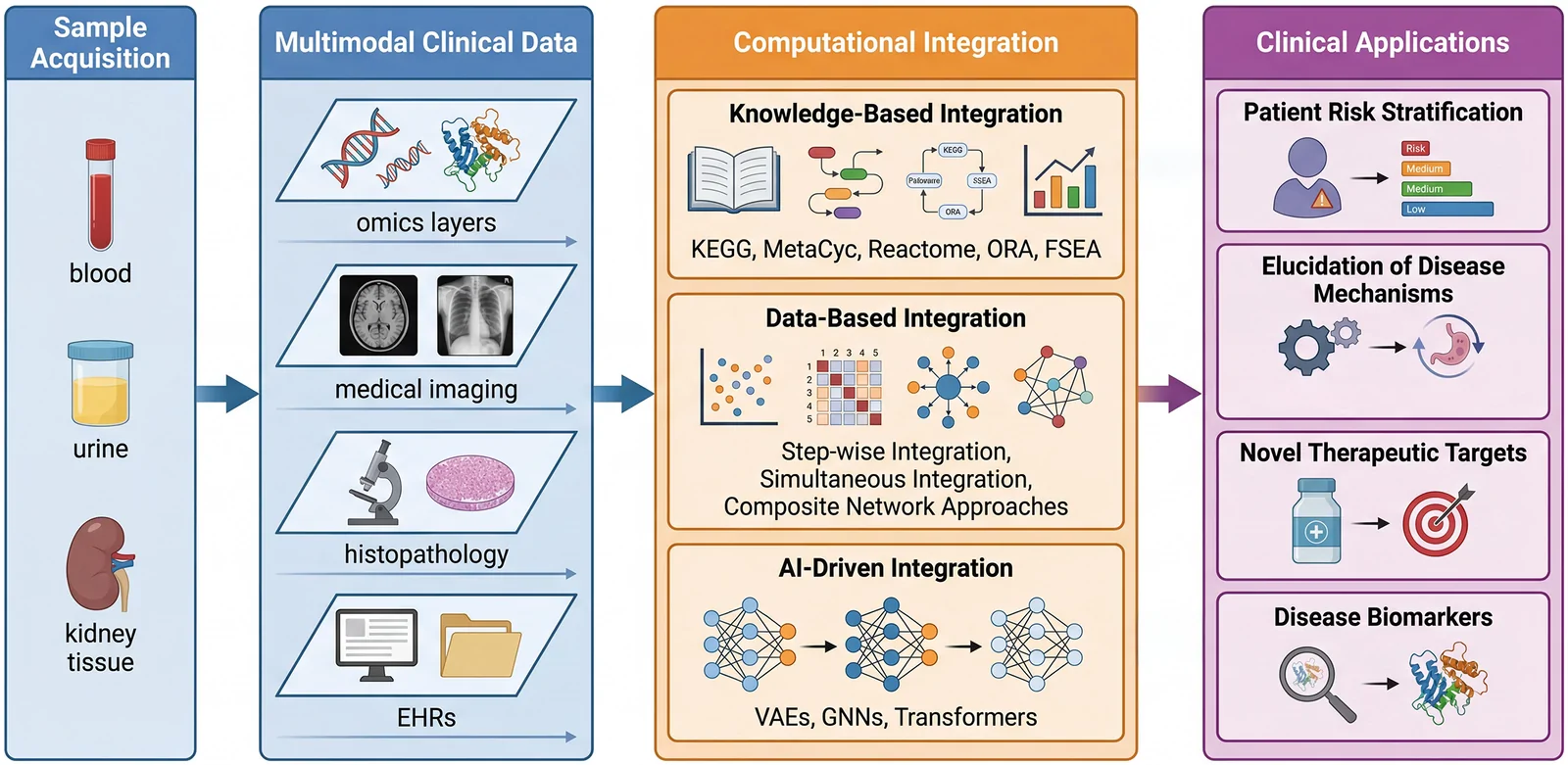
The comprehensive translational pipeline for multimodal clinical data integration. *Note*: This schematic illustrates the end-to-end workflow from sample acquisition to clinical application. Biological specimens (blood, urine and renal tissue) are processed into multimodal clinical data, spanning omics layers, medical imaging, histopathology and EHRs. These data are analysed through a three-pronged computational integration framework: knowledge-based integration, data-based integration and AI-driven integration. This integrated pipeline ultimately facilitates high-impact clinical outcomes, including patient risk stratification, elucidation of disease mechanisms, identification of novel therapeutic targets and discovery of disease biomarkers. EHRs: electronic health records; KEGG: Kyoto Encyclopedia of Genes and Genomes; MetaCyc: Metabolic Pathways Database; ORA: over-representation analysis; FSEA: functional set enrichment analysis; VAEs: variational autoencoders; GNNs: graph neural networks; AI: artificial intelligence. Figure 3. long description.

Recent studies have successfully developed advanced multimodal fusion models, such as DeepOmix-FLEX, that simultaneously incorporate clinical variables, proteomic and metabolomic profiles, and digitized histopathological images. These models achieve an AUC of 0.89 for predicting CKD progression, substantially outperforming traditional models that rely solely on clinical variables (AUCs of 0.72–0.78) (Ref. 164). Furthermore, AI-embedded EHRs enable real-time AKI monitoring and proactive interventions, such as optimizing fluid management and making timely adjustments to nephrotoxic drugs (Ref. 165).

The integration of multi-omics data with medical imaging represents another highly promising frontier. Current studies have found that models integrating multi-organ imaging-derived phenotypes and PRS outperform single-modality models across seven diseases, including heart failure, coronary artery disease and CKD. For example, the integrated model improved the predictive AUC for CKD from 0.53 to 0.67, confirming that multimodal data integration can significantly enhance predictive performance for common diseases (Ref. 166). Importantly, when imaging-derived features are co-registered and analysed alongside spatial transcriptomic or proteomic data, these signals can be anchored to specific molecular pathways, thereby enabling non-invasive inference of intrarenal molecular states.

KPMP and NURTuRE are innovative initiatives that combine clinical, histopathological and multi-omics data to characterize molecular subtypes of kidney diseases and to develop tailored treatment approaches (Ref. 167).

### Liquid biopsy and non-invasive multi-omics monitoring

The shift toward minimally invasive molecular monitoring is transforming nephrology. Liquid biopsy, analysing circulating free DNA, extracellular vesicles and soluble molecules in blood and urine, offers a safe, repeatable and dynamic assessment of kidney health, circumventing the risks and biases of traditional tissue biopsy. In AKI, multicentre studies using liquid-biopsy proteomics and machine learning predicted severe outcomes, with AUCs of 0.87 in the training cohort and 0.76 in the validation cohort. Multi-omics analyses revealed dysregulated cell adhesion and autophagy pathways, with proteins enriched in the juxtamedullary nephron, endothelial cells and podocytes, thereby linking extracellular matrix degradation to pathogenesis (Ref. 168).

In CKD, the NURTuRE cohort study combined kidney tissue transcriptomics with plasma and urinary proteomics and metabolomics to construct a multi-omics network (Ref. 81). Patients with similar clinical phenotypes but different molecular subtypes showed markedly different event-free survival, highlighting the value of multi-omics in better patient stratification. As detection methods improve and standardization advances, liquid biopsy-based multi-omics approaches may complement invasive diagnostics and, in selected settings, reduce reliance on tissue biopsy.

### Toward clinical implementation: Precision diagnosis and individualized therapy

Multi-omics integration aims to enable precision medicine in nephrology, but it demands a well-organized clinical implementation framework. First, analytical validity must be ensured through standardized platforms, harmonized protocols and cross-laboratory reproducibility. Second, clinical validity should be established using large-scale, longitudinal, multicentre cohorts, emphasizing dynamic risk modelling over static, single-time-point associations. Third, clinical utility must be demonstrated by showing that multi-omics-informed strategies improve decision-making and patient outcomes in real-world settings. Integration with EHRs and AI-driven decision support systems is crucial to translate molecular data into actionable outputs. Regulatory frameworks should adapt to rapid technological advances, and international standards are essential for scalability. Cost-effectiveness and risk–benefit analyses should guide implementation, with a focus on high-risk populations to prevent overdiagnosis and excessive healthcare burdens. Collectively, a comprehensive pathway involving standardization, validation, clinical integration and economic evaluation is vital to achieve precision nephrology.

## Conclusion

Multi-omics technologies are transforming kidney disease research by enabling integrated molecular characterization of disease mechanisms, biomarkers and therapeutic targets. However, the next stage of progress should be measured not only by the discovery of molecular signatures, but also by their standardization, validation, interpretability and clinical utility. Broader implementation will require multicentre longitudinal validation, harmonized analytical pipelines, regulatory guidance, cost-effectiveness assessment and prospective evidence demonstrating that omics-guided interventions improve patient outcomes.

Accordingly, the field is transitioning from exploratory discovery toward translational application, although clinical adoption will likely proceed in a stepwise manner with rigorous evaluation at each stage. Near-term applications may focus on biomarker-based risk stratification and disease subclassification, whereas longer-term goals include real-time disease monitoring, mechanism-guided therapies and fully personalized renal care. Achieving these objectives will require close collaboration among nephrologists, molecular biologists, bioinformaticians, data scientists, industry partners and regulatory agencies. With continued technological refinement and prospective clinical validation, multi-omics has the potential to establish a new paradigm for precision nephrology and ultimately improve global kidney health outcomes.

## Long descriptions

### Figure 1. Long description

The workflow is organized into four sequential stages.

1. Specimen Provenance and Molecular Extraction: On the far left, three sources are shown: Blood (vial and D N A helix), Urine (cup and E V s), and Kidney Tissue (organ and biopsy needle). Arrows from these sources converge into a central Molecular Extraction node.

2. Dual-Track Analytical Workflows: The extraction node splits into two paths.

- The top path, In Situ and Tissue-Level Path, shows a kidney leading to a cluster of diverse cells, which then branches into Single-Cell Omics (a cluster plot) and Spatial Omics (a heat-mapped kidney section).

- The bottom path, Conventional Bulk Omics Path, leads from a centrifuge tube to a list of five disciplines: Genomics, Epigenomics, Transcriptomics, Proteomics, and Metabolomics.

3. Multi-omics Data Integration: Both paths feed into a circular integration hub. At the center is a microchip icon. Surrounding it in a clockwise cycle are Bioinformatics (computer server), A I / M L Algorithms (neural network diagram), and Cloud Database (cloud icon).

4. Clinical Translation: The integration hub points to four final outcomes on the right:

- Unravel Disease Heterogeneity: Icons of diseased kidneys and patient triage.

- Discover Novel Biomarkers: A micro-array plate and blood vial.

- Elucidate Molecular Mechanisms: A complex signaling pathway diagram labeled Inflammatory.

- Advance Precision Therapeutics: A patient silhouette next to a monitor and medical equipment.

Navigate back to Figure 1..

### Figure 2. Long description

The workflow begins on the left with a vertical stack titled Multi-omics Data Layers, containing four diamond-shaped icons: Genomics (D N A helix), Transcriptomics (R N A strand), Proteomics (protein structures), and Metabolomics (chemical structures). Arrows lead from these layers into three central integration strategy boxes.

* The top box, KNOWLEDGE-BASED INTEGRATION, shows a K E G G and Pathways network diagram and an open book, leading to an icon of a brain labeled Functional Insights.

* The middle box, DATA-DRIVEN INTEGRATION, is split into Step-wise and Simultaneous approaches. Step-wise shows a sequential flow between omics icons. Simultaneous shows four omics inputs feeding into a single cube containing a summation symbol, network nodes, and a heat map.

* The bottom box, NETWORK-BASED INTEGRATION, displays a complex multi-layer network with nodes connected by lines of varying thickness.

Arrows from these three strategies converge into a vertical panel on the right containing advanced computational methods: Variational Autoencoders (represented by a neural network bottleneck diagram), Graph Neural Networks (a node-link diagram), and Cloud Computing (a server rack icon).

Finally, a downward arrow points to the TRANSLATIONAL IMPACT section at the bottom right, which lists four goals with corresponding icons: Molecular Subtyping (human silhouette), Biomarker Discovery (magnifying glass over D N A), Mechanistic Insight (gears and cells), and Precision Therapy (a pill bottle).

Navigate back to Figure 2..

### Table 1. Long description

The table consists of five columns: Study (year), Disease type, Omics layers, Key findings, and Clinical significance.

* Dumoulin et al. (Ref. 76): Focuses on D K D using single-cell spatial transcriptomics and sn R N A-seq. It identified a B-cell-enriched subgroup and developed a 14-protein plasma biomarker panel for non-invasive detection.

* Liu et al. (Ref. 77): Focuses on C K D using G W A S, bulk R N A-seq, and single-nucleus multiome. It identified 1026 independent loci and provides a Kidney Disease Genetic Scorecard.

* Zhang et al. (Ref. 70): Focuses on A K I using bulk transcriptomics, metabolomics, sc R N A-seq, and spatial transcriptomics. It identified C m p k 1 as a central hub gene and therapeutic target.

* Abedini, A. et al. (Ref. 78): Focuses on C K D using sn R N A-seq, sc R N A-seq, sn A T A C-seq, and spatial transcriptomics. It revealed four distinct microenvironments including the fibrotic microenvironment (F M E).

* Lake et al. (Ref. 71): Focuses on healthy and injured kidneys (A K I or C K D) using sn R N A-seq, spatial transcriptomics, 3 D imaging, and epigenomics. It defined 51 main cell types and 28 injury-altered states.

* Li D et al. (Ref. 79): Focuses on D K D using proteomics and single-cell R N A-seq. It identified A K R 1 A 1 as a molecular hub for cellular dysfunction.

* Li et al. (Ref. 20): Focuses on kidney anatomy using simultaneous single-cell A T A C or R N A sequencing and spatially resolved metabolomics. It identified P L E K H A 1 as a disease marker.

* Wang et al. (Ref. 80): Focuses on Lupus nephritis (L N) using single-cell multiome, spatial transcriptomics, and G W A S. It identified V C A M 1-expressing proximal tubule cells as part of an L N-specific inflammatory niche.

* Castro et al. (Ref. 81): Focuses on C K D using transcriptomics, proteomics, metabolomics, and sn R N A-seq. It discovered two molecularly distinct patient groups with different survival curves.

* Si et al. (Ref. 82): Focuses on C K D using G W A S, T W A S, and proteomics. It identified 32 causal proteins for C K D via Mendelian randomization.

* Huang et al. (Ref. 83): Focuses on A K I using proteomics and metabolomics. It proposed the I C 3 diagnostic model for early screening.

Navigate back to Table 1..

### Table 2. Long description

The table contains 17 rows of biomarkers with the following data:

* C K D dash 273: For C K D or D K D. A 273-peptide urinary classifier for collagen degradation and inflammation. Identifies high-risk individuals with normal albumin. Limitations include high cost and mass spectrometry requirements. Validation: F D A letter of support.

* Cystatin C: For A K I or C K D. Produced by nucleated cells, filtered by glomerulus. Better than creatinine for early A K I. Affected by thyroid and steroids. Validation: In clinical use.

* N G A L: For A K I. Secreted by tubular cells. Warns 24 to 48 hours before creatinine rise. Elevated in systemic infection. Validation: F D A approved.

* K I M dash 1: For A K I or C K D. Transmembrane glycoprotein in proximal tubules. Predicts C K D progression. Also upregulated in R C C. Validation: Clinical validation phase.

* su P A R: For A K I or C K D. Soluble receptor released during inflammation. Predicts A K I onset and C K D progression. Poor specificity. Validation: Clinical validation phase.

* F G F dash 23: For C K D. Phosphate-regulating hormone. Early marker of C K D dash M B D. Influenced by iron deficiency and inflammation. Validation: Clinical validation phase.

* D K K 3: For C K D, A K I, or Alport syndrome. Tubule-derived Wnt antagonist. Marker of fibrosis. High E L I S A cost. Validation: Clinical validation phase.

* U M O D (Uromodulin): For C K D or A D T K D. Reflects tubular mass. Low levels indicate A K I risk. Affected by hydration status. Validation: Widely recognized for genetic diagnosis.

* T I M P dash 2 dot I G F B P 7: For A K I. Reflects G 1 cell cycle arrest. High specificity for severe A K I. Applicable to I C U populations. Validation: F D A approved (NephroCheck).

* alpha 1 dash Microglobulin: For C K D or D K D. Low-molecular-weight protein. Early marker of tubular injury. Affected by liver function. Validation: In clinical use.

* L dash F A B P: For A K I, C K D, or D K D. Released during oxidative stress. Predicts A K I after cardiac surgery. Not kidney-specific. Validation: Approved in Japan.

* Urinary E G F: For C K D, A K I, or D K D. Reflects functional tubular mass. Low levels associated with function decline. Requires standardized detection. Validation: Clinical validation phase.

* W F D C 2: For C K D or Fibrosis. Serine protease inhibitor. Potential therapeutic target. Limited clinical data. Validation: F D A authorized for KidneyIntelX.

* G D F 15: For A K I or C K D. T G F dash beta superfamily member. Associated with mortality risk. Not kidney-specific. Validation: Clinical research phase.

* Urinary C K 20: For A K I to C K D transition. Cytokeratin 20 upregulated after injury. Predicts major adverse kidney events. Needs generalizability validation. Validation: Clinical research phase.

* A K R 1 A 1: For D K D. Aldo-keto reductase family member. Protective role in African American patients. Limited ethnic group validation. Validation: Clinical research phase.

* A N G P T 2: For A K I and C K D. Endothelial activation marker. Correlates with sepsis-associated A K I severity. Not kidney-specific. Validation: Clinical research phase.

Navigate back to Table 2..

### Figure 3. Long description

The flowchart consists of four vertical panels connected by horizontal arrows.

1. Sample Acquisition (Leftmost panel): Contains icons for blood (test tube), urine (specimen cup), and kidney tissue (anatomical kidney).

2. Multimodal Clinical Data (Second panel): Four horizontal layers showing:

- Omics layers: D N A helix and protein structure icons.

- Medical imaging: M R I brain scan and X-ray chest icons.

- Histopathology: Microscope and petri dish icons.

- E H Rs: Computer monitor and folder icons.

3. Computational Integration (Third panel): Three stacked boxes:

- Knowledge-Based Integration: Icons for a book, metabolic pathways, and a circular network including KEGG, MetaCyc, Reactome, O R A, and F S E A.

- Data-Based Integration: Icons for scatter plots, heatmaps, and network graphs. Text includes Step-wise Integration, Simultaneous Integration, and Composite Network Approaches.

- A I-Driven Integration: Three neural network diagrams connected by arrows. Text includes V A Es, G N Ns, and Transformers.

4. Clinical Applications (Rightmost panel): Four outcome boxes:

- Patient Risk Stratification: Icon of a person pointing to a risk scale (High, Medium, Low).

- Elucidation of Disease Mechanisms: Gear icons pointing to a stomach diagram.

- Novel Therapeutic Targets: Medicine bottle icon pointing to a bullseye target.

- Disease Biomarkers: Magnifying glass icon over protein structures.

Navigate back to Figure 3..

## References

[1] Foreman KJ, Marquez N, Dolgert A, Fukutaki K, Fullman N, McGaughey M, Pletcher MA, Smith AE, Tang K, Yuan C-W, et al. (2018) Forecasting life expectancy, years of life lost, and all-cause and cause-specific mortality for 250 causes of death: Reference and alternative scenarios for 2016-40 for 195 countries and territories. *Lancet (London, England)*, 392(10159):2052–2090. 10.1016/S0140-6736(18)31694-5 PMC6227505 30340847

[2] Mark PB, Stafford LK, Grams ME, Aalruz H, Abd ElHafeez S, Abdelgalil AA, Abdulkader RS, Abeywickrama HM, Abiodun OO, Abramov D, et al. (2025) Global, regional, and national burden of chronic kidney disease in adults, 1990–2023, and its attributable risk factors: A systematic analysis for the global burden of disease study 2023. The Lancet 406(10518), 2461–2482. 10.1016/S0140-6736(25)01853-7. 41213283

[3] Wang L, Xu X, Zhang M, Hu C, Zhang X, Li C, Nie S, Huang Z, Zhao Z, Hou FF and Zhou M (2023) Prevalence of chronic kidney disease in China: Results from the sixth China chronic disease and risk factor surveillance. JAMA Internal Medicine 183(4), 298–310. 10.1001/jamainternmed.2022.6817. PMC9941971 36804760

[4] AlEmam SA, Al-Shatnawi SF and Sheikh Ghadzi SM (2026) Recurrent acute kidney injury in hospitalized adults: Incidence, risk factors, and outcomes-a systematic review and meta-analysis. The American Journal of the Medical Sciences 371(6), 528–536. 10.1016/j.amjms.2026.03.010. 41833689

[5] (2020) Precision medicine in nephrology. Nature Reviews Nephrology 16(11), 615. 10.1038/s41581-020-00360-9. 33028956

[6] Hull KL, Adenwalla SF, Topham P and Graham-Brown MP (2022) Indications and considerations for kidney biopsy: An overview of clinical considerations for the non-specialist. Clinical Medicine (London, England) 22(1), 34–40. 10.7861/clinmed.2021-0472. PMC8813013 34921054

[7] Saliba A and Feng Y (2024) Multi-omics integration in nephrology: Advances, challenges, and future directions. Seminars in Nephrology 44(6), 151584. 10.1016/j.semnephrol.2025.151584. PMC12278776 40216576

[8] Delrue C, Speeckaert R and Speeckaert MM (2026) From GWAS signals to causal genes in chronic kidney disease. Current Issues in Molecular Biology 48(2). 10.3390/cimb48020148. PMC12939580 41751412

[9] Aihara S and Muto Y (2025) Single-cell epigenetics and multiomics analysis in kidney research. Clinical and Experimental Nephrology 29(9), 1139–1149. 10.1007/s10157-025-02679-8. PMC12441082 40281349

[10] Liu X, Shi J, Jiao Y, An J, Tian J, Yang Y and Zhuo L (2024) Integrated multi-omics with machine learning to uncover the intricacies of kidney disease. Briefings in Bioinformatics 25(5). 10.1093/bib/bbae364. PMC11289682 39082652

[11] Alobaidi S (2025) Emerging biomarkers and advanced diagnostics in chronic kidney disease: Early detection through multi-omics and AI. Diagnostics (Basel) 15(10). 10.3390/diagnostics15101225. PMC12110191 40428218

[12] Gorman BL, Shafer CC, Ragi N, Sharma K, Neumann EK and Anderton CR (2025) Imaging and spatially resolved mass spectrometry applications in nephrology. Nature Reviews Nephrology 21(6), 399–416. 10.1038/s41581-025-00946-1. 40148534

[13] Sharma K, Hansen J, Susztak K, Eberlin L, Anderton CR, Alexandrov T and Iyengar R (2026) Spatial metabolomics and multiomics integration for breakthroughs in precision medicine for kidney disease. Nature Reviews Nephrology 22(2), 152–164. 10.1038/s41581-025-01007-3. PMC13264805 41068470

[14] Pandey S (2025) Metabolomics for the identification of biomarkers in kidney diseases. Nano 9(2), 110–120. 10.7150/ntno.108320. PMC11980039 40212952

[15] Garoufis M, Sakkou SF, Kostara CE, Bairaktari E and Tsimihodimos V (2026) Metabolomics for preclinical detection of diabetic kidney disease: A comprehensive review. International Journal of Molecular Sciences 27(2). 10.3390/ijms27020998. PMC12842068 41596641

[16] Stanzick KJ, Li Y, Schlosser P, Gorski M, Wuttke M, Thomas LF, Rasheed H, Rowan BX, Graham SE, Vanderweff BR, Patil SB, VA Million Veteran Program, Robinson-Cohen C, Gaziano JM, O’Donnell CJ, Willer CJ, Hallan S, Åsvold BO, Gessner A, Hung AM, Pattaro C, Köttgen A, Stark KJ, Heid IM and Winkler TW (2021) Discovery and prioritization of variants and genes for kidney function in >1.2 million individuals. Nature Communications 12(1), 4350. 10.1038/s41467-021-24491-0. PMC8285412 34272381

[17] Hellwege JN, Velez Edwards DR, Giri A, Qiu C, Park J, Torstenson ES, Keaton JM, Wilson OD, Robinson-Cohen C, Chung CP, Roumie CL, Klarin D, Damrauer SM, DuVall SL, Siew E, Akwo EA, Wuttke M, Gorski M, Li M, Li Y, Gaziano JM, Wilson PWF, Tsao PS, O’Donnell CJ, Kovesdy CP, Pattaro C, Köttgen A, Susztak K, Edwards TL and Hung AM (2019) Mapping eGFR loci to the renal transcriptome and phenome in the VA million veteran program. Nature Communications 10(1), 3842. 10.1038/s41467-019-11704-w. PMC6710266 31451708

[18] Khan A and Kiryluk K (2025) Polygenic scores and their applications in kidney disease. Nature Reviews Nephrology 21(1), 24–38. 10.1038/s41581-024-00886-2. 39271761

[19] Ding H, Zhang L, Yang Q, Zhang X, and Li X (2021) Epigenetics in kidney diseases. Advances in Clinical Chemistry, 104, 233–297. 10.1016/bs.acc.2020.09.005. PMC9322755 34462056

[20] Li H, Li D, Ledru N, Xuanyuan Q, Wu H, Asthana A, Byers LN, Tullius SG, Orlando G, Waikar SS, et al. (2024) Transcriptomic, epigenomic, and spatial metabolomic cell profiling redefines regional human kidney anatomy. Cell Metabolism 36(5), 1105–1125.e10. 10.1016/j.cmet.2024.02.015 PMC11081846 38513647

[21] Guo C, Dong G, Liang X and Dong Z (2019) Epigenetic regulation in AKI and kidney repair: Mechanisms and therapeutic implications. Nature Reviews Nephrology 15(4), 220–239. 10.1038/s41581-018-0103-6. PMC7866490 30651611

[22] Tan R-Z, Jia J, Li T, Wang L and Kantawong F (2024) A systematic review of epigenetic interplay in kidney diseases: Crosstalk between long noncoding RNAs and methylation, acetylation of chromatin and histone. Biomedicine & pharmacotherapy = Biomedecine & pharmacotherapie 176, 116922. 10.1016/j.biopha.2024.116922. 38870627

[23] Craciun FL, Bijol V, Ajay AK, Rao P, Kumar RK, Hutchinson J, Hofmann O, Joshi N, Luyendyk JP, Kusebauch U, Moss CL, Srivastava A, Himmelfarb J, Waikar SS, Moritz RL and Vaidya VS (2016) RNA sequencing identifies novel translational biomarkers of kidney fibrosis. Journal of the American Society of Nephrology 27(6), 1702–1713. 10.1681/ASN.2015020225. PMC4884105 26449608

[24] Lake BB, Chen S, Hoshi M, Plongthongkum N, Salamon D, Knoten A, Vijayan A, Venkatesh R, Kim EH, Gao D, Gaut J, Zhang K and Jain S (2019) A single-nucleus RNA-sequencing pipeline to decipher the molecular anatomy and pathophysiology of human kidneys. Nature Communications 10(1), 2832. 10.1038/s41467-019-10861-2. PMC6597610 31249312

[25] Pliner HA, Packer JS, McFaline-Figueroa JL, Cusanovich DA, Daza RM, Aghamirzaie D, Srivatsan S, Qiu X, Jackson D, Minkina A, Adey AC, Steemers FJ, Shendure J and Trapnell C (2018) Cicero predicts cis-regulatory DNA interactions from single-cell chromatin accessibility data. Molecular Cell 71(5), 858–871.e8. 10.1016/j.molcel.2018.06.044. PMC6582963 30078726

[26] Schep AN, Wu B, Buenrostro JD and Greenleaf WJ (2017) chromVAR: Inferring transcription-factor-associated accessibility from single-cell epigenomic data. Nature Methods 14(10), 975–978. 10.1038/nmeth.4401. PMC5623146 28825706

[27] Mosterd CM, Verhaar BJH, van den Born BH, Nieuwdorp M and van Raalte DH (2025) Plasma and urine metabolites associated with nondiabetic chronic kidney disease: The HELIUS study. Kidney Medicine 7(7), 101009. 10.1016/j.xkme.2025.101009. PMC12221752 40613013

[28] Beck LH, Bonegio RGB, Lambeau G, Beck DM, Powell DW, Cummins TD, Klein JB and Salant DJ (2009) M-type phospholipase A2 receptor as target antigen in idiopathic membranous nephropathy. The New England Journal of Medicine 361(1), 11–21. 10.1056/NEJMoa0810457. PMC2762083 19571279

[29] Sethi S (2021) New ’antigens’ in membranous nephropathy. Journal of the American Society of Nephrology: JASN 32(2), 268–278. 10.1681/ASN.2020071082. PMC8054892 33380523

[30] Wang Y-N, Ma S-X, Chen Y-Y, Chen L, Liu B-L, Liu Q-Q, Zhao Y-Y (2019) Chronic kidney disease: Biomarker diagnosis to therapeutic targets. Clinica Chimica Acta; International Journal of Clinical Chemistry 499:54–63. 10.1016/j.cca.2019.08.030. 31476302

[31] Zhu S, Zhang F, Shen A-W, Sun B, Xia T-Y, Chen W-S, Tao X and Yu S-Q (2021) Metabolomics evaluation of patients with stage 5 chronic kidney disease before dialysis, maintenance Hemodialysis, and peritoneal dialysis. Frontiers in Physiology 11, 630646. 10.3389/fphys.2020.630646. PMC7855177 33551851

[32] Kalim S and Rhee EP (2017) An overview of renal metabolomics. Kidney International 91(1), 61–69. 10.1016/j.kint.2016.08.021. PMC5380230 27692817

[33] Schmidt IM and Rhee EP (2026) Interpreting metabolomics and proteomics in kidney disease: A practical guide. American Journal of Kidney Diseases 87(1), 129–133. 10.1053/j.ajkd.2025.08.014. 41192615

[34] Cabała S and Herosimczyk A (2025) Diet-induced proteomic and metabolomic signatures in chronic kidney disease: A precision nutrition approach. Metabolites 15(3). 10.3390/metabo15030211. PMC11943711 40137175

[35] Vandereyken K, Sifrim A, Thienpont B and Voet T (2023) Methods and applications for single-cell and spatial multi-omics. Nature Reviews Genetics 24(8), 494–515. 10.1038/s41576-023-00580-2. PMC9979144 36864178

[36] Lee Y, Lee M, Shin Y, Kim K and Kim T (2025) Spatial omics in clinical research: A comprehensive review of technologies and guidelines for applications. International Journal of Molecular Sciences 26(9), 3949. 10.3390/ijms26093949. PMC12071594 40362187

[37] Gorman BL and Lukowski JK (2024) Spatial metabolomics and Lipidomics in kidney disease. Seminars in Nephrology 44(6), 151582. 10.1016/j.semnephrol.2025.151582. 40234137

[38] Liu X, Peng T, Xu M, Lin S, Hu B, Chu T, Liu B, Xu Y, Ding W, Li L, Cao C and Wu P (2024) Spatial multi-omics: Deciphering technological landscape of integration of multi-omics and its applications. Journal of Hematology & Oncology 17(1), 72. 10.1186/s13045-024-01596-9. PMC11344930 39182134

[39] Klomp LS, Mavrommatis L, Arcolino FO, Lantermans HC, Levtchenko E, Kuppe C and Westland R (2026) Dissecting Normal and abnormal human kidney development using multiomics. Journal of the American Society of Nephrology 37(3), 585–595. 10.1681/ASN.0000000951. PMC12935381 41212627

[40] Jendoubi T (2021) Approaches to integrating metabolomics and multi-omics data: A primer. Metabolites 11(3). 10.3390/metabo11030184. PMC8003953 33801081

[41] Wörheide MA, Krumsiek J, Kastenmüller G and Arnold M (2021) Multi-omics integration in biomedical research - a metabolomics-centric review. Analytica Chimica Acta 1141, 144–162. 10.1016/j.aca.2020.10.038. PMC7701361 33248648

[42] Kanehisa M and Goto S (2000) KEGG: Kyoto encyclopedia of genes and genomes. Nucleic Acids Research 28(1), 27–30. 10.1093/nar/28.1.27. PMC102409 10592173

[43] Karp PD, Riley M, Paley SM and Pellegrini-Toole A (2002) The MetaCyc database. Nucleic Acids Research 30(1), 59–61. 10.1093/nar/30.1.59. PMC99148 11752254

[44] Joshi-Tope G, Vastrik I, Gopinath GR, Matthews L, Schmidt E, Gillespie M, D’Eustachio P, Jassal B, Lewis S, Wu G, et al. (2003) The genome knowledgebase: A resource for biologists and bioinformaticists. Cold Spring Harbor Symposia on Quantitative Biology 68, 237–244. 10.1101/sqb.2003.68.237. 15338623

[45] Khatri P, Sirota M and Butte AJ (2012) Ten years of pathway analysis: Current approaches and outstanding challenges. PLoS Computational Biology 8(2), e1002375. 10.1371/journal.pcbi.1002375. PMC3285573 22383865

[46] Xia J and Wishart DS (2010) MSEA: A web-based tool to identify biologically meaningful patterns in quantitative metabolomic data. Nucleic Acids Research 38(Web Server), W71–W77. 10.1093/nar/gkq329. PMC2896187 20457745

[47] Subramanian A, Tamayo P, Mootha VK, Mukherjee S, Ebert BL, Gillette MA, Paulovich A, Pomeroy SL, Golub TR, Lander ES and Mesirov JP (2005) Gene set enrichment analysis: A knowledge-based approach for interpreting genome-wide expression profiles. Proceedings of the National Academy of Sciences 102(43), 15545–15550. 10.1073/pnas.0506580102. PMC1239896 16199517

[48] Rokach L (2010) Ensemble-based classifiers. Artificial Intelligence Review 33(1–2), 1–39. 10.1007/s10462-009-9124-7.

[49] Ritchie MD, Holzinger ER, Li R, Pendergrass SA and Kim D (2015) Methods of integrating data to uncover genotype–phenotype interactions. Nature Reviews Genetics 16(2), 85–97. 10.1038/nrg3868. 25582081

[50] Hirschhorn JN and Daly MJ (2005) Genome-wide association studies for common diseases and complex traits. Nature Reviews Genetics 6(2), 95–108. 10.1038/nrg1521. 15716906

[51] Sun S (2013) A survey of multi-view machine learning. Neural Computing and Applications 23(7–8), 2031–2038. 10.1007/s00521-013-1362-6.

[52] Trygg J and Wold S (2003) O2-PLS, a two-block (X–Y) latent variable regression (LVR) method with an integral OSC filter. Journal of Chemometrics 17(1), 53–64. 10.1002/cem.775.

[53] Löfstedt T and Trygg J (2011) OnPLS—A novel multiblock method for the modelling of predictive and orthogonal variation. Journal of Chemometrics 25(8), 441–455. 10.1002/cem.1388.

[54] Tenenhaus A and Tenenhaus M (2011) Regularized generalized canonical correlation analysis. Psychometrika 76(2), 257–284. 10.1007/s11336-011-9206-8. 28536930

[55] Singh A, Shannon CP, Gautier B, Rohart F, Vacher M, Tebbutt SJ and Lê Cao K-A (2019) DIABLO: An integrative approach for identifying key molecular drivers from multi-omics assays. Bioinformatics 35(17), 3055–3062. 10.1093/bioinformatics/bty1054. PMC6735831 30657866

[56] Vidal M, Cusick Michael E and Barabási A-L (2011) Interactome networks and human disease. Cell 144(6), 986–998. 10.1016/j.cell.2011.02.016. PMC3102045 21414488

[57] Sügis E, Dauvillier J, Leontjeva A, Adler P, Hindie V, Moncion T, Collura V, Daudin R, Loe-Mie Y, Herault Y, Lambert JC, Hermjakob H, Pupko T, Rain JC, Xenarios I, Vilo J, Simonneau M and Peterson H (2019) HENA, heterogeneous network-based data set for Alzheimer’s disease. Scientific Data 6(1), 151. 10.1038/s41597-019-0152-0. PMC6694132 31413325

[58] Kamburov A, Wierling C, Lehrach H, Herwig R (2009) ConsensusPathDB—A database for integrating human functional interaction networks. Nucleic Acids Research 37(suppl_1), D623–D628. 10.1093/nar/gkn698. PMC2686562 18940869

[59] Zhou G and Xia J (2019) Using OmicsNet for network integration and 3D visualization. Current Protocols in Bioinformatics 65(1), e69. 10.1002/cpbi.69. 30556956

[60] Sabanayagam C, Banu R, Lim C, Tham YC, Cheng CY, Tan G, Ekinci E, Sheng B, McKay G, Shaw JE, Matsushita K, Tangri N, Choo J and Wong TY (2025) Artificial intelligence in chronic kidney disease management: A scoping review. Theranostics 15(10), 4566–4578. 10.7150/thno.108552. PMC11984408 40225559

[61] Tian T, Zhang J, Lin X, Wei Z and Hakonarson H (2024) Dependency-aware deep generative models for multitasking analysis of spatial omics data. Nature Methods 21(8), 1501–1513. 10.1038/s41592-024-02257-y. PMC12410143 38783067

[62] Zhang Z, Wang M, Zhang X, Dai R, Wang Z, Lei L, Li Z, Han K, Wang Z, Shi C and Guo Q (2025) SpaOmicsVAE: A deep learning framework for integrative analysis of spatial multi-omics data. Computer Methods and Programs in Biomedicine 271, 109032. 10.1016/j.cmpb.2025.109032. 40848558

[63] Milite S, Caravagna G and Sottoriva A (2025) MIDAA: Deep archetypal analysis for interpretable multi-omic data integration based on biological principles. Genome Biology 26(1), 90. 10.1186/s13059-025-03530-9. PMC11980162 40200293

[64] Ewald JD, Zhou G, Lu Y, Kolic J, Ellis C, Johnson JD, Macdonald PE and Xia J (2024) Web-based multi-omics integration using the analyst software suite. Nature Protocols 19(5), 1467–1497. 10.1038/s41596-023-00950-4. 38355833

[65] Li C, Ziyadeh E, Sharma Y, Dumoulin B, Levinsohn J, Ha E, Pan S, Rao V, Subramaniyam M, Szegedy M, et al. (2025) Nephrobase cell+: Multimodal single-cell foundation model for decoding kidney biology. arXiv.

[66] Li S and Susztak K (2025) Mitochondrial dysfunction has a central role in diabetic kidney disease. Nature Reviews Nephrology 21(2), 77–78. 10.1038/s41581-024-00919-w. 39681602

[67] Miguel V and Kramann R (2023) Metabolic reprogramming heterogeneity in chronic kidney disease. FEBS Open Bio 13(7), 1154–1163. 10.1002/2211-5463.13568. PMC10315765 36723270

[68] Cao M, Zhao X, Xia F, Shi M, Zhao D, Li L and Jiang H (2025) Mitochondrial dysfunction and metabolic reprogramming in acute kidney injury: Mechanisms, therapeutic advances, and clinical challenges. Frontiers in Physiology 16, 1623500. 10.3389/fphys.2025.1623500. PMC12364819 40843128

[69] Moon Y, Hong J, Chung J and Baek J-H (2026) Macrophage plasticity and regulatory networks during the transition from inflammation to fibrosis in the kidney. Life 16(1), 52. 10.3390/life16010052. PMC12842797 41598208

[70] Zhang H, Zhang G, Wang B, Xiong F, Ji X and Wang C (2025) Integrated multi-omics profiling identifies Cmpk1 as a monocyte-specific therapeutic target for renal ischemia-reperfusion injury. Life Sciences 380, 123924. 10.1016/j.lfs.2025.123924. 40848967

[71] Lake BB, Menon R, Winfree S, Hu Q, Melo Ferreira R, Kalhor K, Barwinska D, Otto EA, Ferkowicz M, Diep D, et al. (2023) An atlas of healthy and injured cell states and niches in the human kidney. Nature 619(7970), 585–594. 10.1038/s41586-023-05769-3. PMC10356613 37468583

[72] Legouis D, Rinaldi A, Malpetti D, Arnoux G, Verissimo T, Faivre A, Mangili F, Rinaldi A, Ruinelli L, Pugin J, Moll S, Clivio L, Bolis M, de Seigneux S, Azzimonti L and Cippà PE (2024) A transfer learning framework to elucidate the clinical relevance of altered proximal tubule cell states in kidney disease. iScience 27(3), 109271. 10.1016/j.isci.2024.109271. PMC10937833 38487013

[73] Kirita Y, Wu H, Uchimura K, Wilson PC and Humphreys BD (2020) Cell profiling of mouse acute kidney injury reveals conserved cellular responses to injury. Proceedings of the National Academy of Sciences of the United States of America 117(27), 15874–15883. 10.1073/pnas.2005477117. PMC7355049 32571916

[74] Reck M, Baird DP, Veizades S, Sutherland C, Bell RMB, Hur H, Cairns C, Janas PP, Campbell R, Nam A, Yang W, Schurman N, Williams C, O’Sullivan E, Beniazza M, Corsinotti A, Bellamy C, Hughes J, Laird A, Denby L, Chandra T, Ferenbach DA and Conway BR (2025) Multiomic analysis of human kidney disease identifies a tractable inflammatory and pro-fibrotic tubular cell phenotype. Nature Communications 16(1), 4745. 10.1038/s41467-025-59997-4. PMC12095627 40399382

[75] Martinez Leon V and Susztak K (2025) Uncovering the metabolic architecture of CKD through integrated metabolomics, transcriptomics, and genetics of human kidney tissue. Journal of the American Society of Nephrology 36(10S). 10.1681/ASN.2025smp9hp1x.

[76] Dumoulin B, Levinsohn J, Klötzer KA, Li C, Mao L, Ha E, Mohandes S, Nguyen T, Paruzzo L, Hirohama D, et al. (2026) Spatial atlas of diabetic kidney disease reveals a B cell-rich subgroup. Nature 653(8116), 1158–1169. 10.1038/s41586-026-10363-4. PMC13216073 42056516

[77] Liu H, Abedini A, Ha E, Ma Z, Sheng X, Dumoulin B, Qiu C, Aranyi T, Li S, Dittrich N, et al. (2025) Kidney multiome-based genetic scorecard reveals convergent coding and regulatory variants. Science 387(6734), eadp4753. 10.1126/science.adp4753. PMC12013656 39913582

[78] Abedini A, Levinsohn J, Klötzer KA, Dumoulin B, Ma Z, Frederick J, Dhillon P, Balzer MS, Shrestha R, Liu H, Vitale S, Bergeson AM, Devalaraja-Narashimha K, Grandi P, Bhattacharyya T, Hu E, Pullen SS, Boustany-Kari CM, Guarnieri P, Karihaloo A, Traum D, Yan H, Coleman K, Palmer M, Sarov-Blat L, Morton L, Hunter CA, Kaestner KH, Li M and Susztak K (2024) Single-cell multi-omic and spatial profiling of human kidneys implicates the fibrotic microenvironment in kidney disease progression. Nature Genetics 56(8), 1712–1724. 10.1038/s41588-024-01802-x. PMC11592391 39048792

[79] Li D, Hsu F-C, Palmer ND, Liu L, Choi YA, Murea M, Parks JS, Bowden DW, Freedman BI and Ma L (2024) Multiomics analyses identify AKR1A1 as a biomarker for diabetic kidney disease. Diabetes 73(7), 1188–1195. 10.2337/db23-0540. PMC11189831 38394643

[80] Wang J, Zheng A, Liu N, Tan Z, Shi Y, Ma T, Luo S, Zhu L, Zhou Z, Yuan F, Li T, Gong Y, Fang J, Liu L, Zhang X, Bae SC, Terao C, Chen Z, Li X, Wang G, Qu K and Guo C (2025) Integrative spatial multiomics analysis reveals regulatory mechanisms of VCAM1(+) proximal tubule cells in lupus nephritis. Annals of the Rheumatic Diseases 84(12), 2021–2033. 10.1016/j.ard.2025.08.015. 40973516

[81] Castro R, Rerra A-I, Urbanke H, Mosblech A, Skroblin P, Radresa O, Andag U and Pinkosky SL (2025) Integrative multiomics network analysis reveals distinct molecular subtypes in CKD. Journal of the American Society of Nephrology 36(10S). 10.1681/ASN.2025wvg981e3.

[82] Si S, Liu H, Xu L and Zhan S (2024) Identification of novel therapeutic targets for chronic kidney disease and kidney function by integrating multi-omics proteome with transcriptome. Genome Medicine 16(1), 84. 10.1186/s13073-024-01356-x. PMC11186236 38898508

[83] Huang P, Liu Y, Li Y, Xin Y, Nan C, Luo Y, Feng Y, Jin N, Peng Y, Wang D, Zhou Y, Luan F, Wang X, Wang X, Li H, Zhou Y, Zhang W, Liu Y, Yuan M, Zhang Y, Song Y, Xiao Y, Shen L, Yu K, Zhao M, Cheng L and Wang C (2025) Metabolomics- and proteomics-based multi-omics integration reveals early metabolite alterations in sepsis-associated acute kidney injury. BMC Medicine 23(1), 79. 10.1186/s12916-025-03920-7. PMC11818193 39934788

[84] Doke T and Susztak K (2022) The multifaceted role of kidney tubule mitochondrial dysfunction in kidney disease development. Trends in Cell Biology 32(10), 841–853. 10.1016/j.tcb.2022.03.012. PMC9464682 35473814

[85] Pavlović N, Križanac M, Kumrić M, Vukojević K and Božić J (2025) Mitochondrial dysfunction: The silent catalyst of kidney disease progression. Cells 14(11). 10.3390/cells14110794. PMC12153900 40497970

[86] Cuevas-Delgado P, Miguel V, Rupérez FJ, Lamas S and Barbas C (2023) Impact of renal tubular Cpt1a overexpression on the kidney metabolome in the folic acid-induced fibrosis mouse model. Frontiers in Molecular Biosciences 10, 1161036. 10.3389/fmolb.2023.1161036. PMC10291237 37377862

[87] Miguel V, Tituaña J, Herrero JI, Herrero L, Serra D, Cuevas P, Barbas C, Puyol DR, Márquez-Expósito L, Ruiz-Ortega M, Castillo C, Sheng X, Susztak K, Ruiz-Canela M, Salas-Salvadó J, González MAM, Ortega S, Ramos R and Lamas S (2021) Renal tubule Cpt1a overexpression protects from kidney fibrosis by restoring mitochondrial homeostasis. The Journal of Clinical Investigation 131(5). 10.1172/JCI140695. PMC7919728 33465052

[88] Mitrofanova A, Merscher S and Fornoni A (2023) Kidney lipid dysmetabolism and lipid droplet accumulation in chronic kidney disease. Nature Reviews Nephrology 19(10), 629–645. 10.1038/s41581-023-00741-w. https://doi.org/10.1038/s41581-023-00741-w. PMC12926870 37500941

[89] Liu P, Peng L, Zhang H, Tang PM, Zhao T, Yan M, Zhao H, Huang X, Lan H and Li P (2018) Tangshen formula attenuates diabetic nephropathy by promoting ABCA1-mediated renal cholesterol efflux in db/db mice. Frontiers in Physiology 9, 343. 10.3389/fphys.2018.00343. PMC5897509 29681863

[90] Wang XX, Jiang T, Shen Y, Adorini L, Pruzanski M, Gonzalez FJ, Scherzer P, Lewis L, Miyazaki-Anzai S and Levi M (2009) The farnesoid X receptor modulates renal lipid metabolism and diet-induced renal inflammation, fibrosis, and proteinuria. American Journal of Physiology 297(6), F1587–F1596. 10.1152/ajprenal.00404.2009. PMC2801344 19776172

[91] Wu W, Lan W, Jiao X, Wang K, Deng Y, Chen R, Zeng R, and Li J (2025) Pyroptosis in sepsis-associated acute kidney injury: Mechanisms and therapeutic perspectives. Critical Care (London, UK) 29(1), 168, 10.1186/s13054-025-05329-3. PMC12020238 40270016

[92] Chung KW, Dhillon P, Huang S, Sheng X, Shrestha R, Qiu C, Kaufman BA, Park J, Pei L, Baur J, et al. (2019) Mitochondrial damage and activation of the STING pathway Lead to renal inflammation and fibrosis. Cell Metabolism 30(4), 784–799.e5. 10.1016/j.cmet.2019.08.003 PMC7054893 31474566

[93] Liang J and Wan C (2026) Chromatin remodeling and epigenetic regulation in chronic kidney disease. Frontiers in Genetics 17, 1781322. 10.3389/fgene.2026.1781322. PMC12844562 41607591

[94] Eustermann S, Patel AB, Hopfner KP, He Y and Korber P (2024) Energy-driven genome regulation by ATP-dependent chromatin remodellers. Nature Reviews Molecular Cell Biology 25(4), 309–332. 10.1038/s41580-023-00683-y. PMC12303036 38081975

[95] Gisch DL, Brennan M, Lake BB, Basta J, Keller MS, Melo Ferreira R, Akilesh S, Ghag R, Lu C, Cheng YH, et al. (2024) The chromatin landscape of healthy and injured cell types in the human kidney. Nature Communications 15(1), 433. 10.1038/s41467-023-44467-6. PMC10781985 38199997

[96] Mimura I, Chen Z and Natarajan R (2025) Epigenetic alterations and memory: Key players in the development/progression of chronic kidney disease promoted by acute kidney injury and diabetes. Kidney International 107(3), 434–456. 10.1016/j.kint.2024.10.031. 39725223

[97] Sasaki K and Masaki T (2025) Epigenetic histone modifications in kidney disease and epigenetic memory. Clinical and Experimental Nephrology 29(9), 1129–1138. 10.1007/s10157-025-02668-x. PMC12441107 40186651

[98] Ledru N, Wilson PC, Muto Y, Yoshimura Y, Wu H, Li D, Asthana A, Tullius SG, Waikar SS, Orlando G and Humphreys BD (2024) Predicting proximal tubule failed repair drivers through regularized regression analysis of single cell multiomic sequencing. Nature Communications 15(1), 1291. 10.1038/s41467-024-45706-0. PMC10861555 38347009

[99] Xuanyuan Q, Wu H, Sundaramoorthi H, Isnard P, Chen C, Rahmani W and Humphreys BD (2025) Multimodal spatial transcriptomic characterization of mouse kidney injury and repair. Nature Communications 16(1), 7567. 10.1038/s41467-025-62599-9. PMC12354736 40813851

[100] Jiang CH, Zhang SJ, Li P, Miao H and Zhao YY (2025) Natural products targeting TGF-β/Smad signaling in renal fibrosis: Multiomics-based novel molecular mechanisms and therapeutic strategies. Phytomedicine 148, 157496. 10.1016/j.phymed.2025.157496. 41202381

[101] Gottlieb ER, Estiverne C, Tolan NV, Melanson SEF and Mendu ML (2023) Estimated GFR with cystatin C and creatinine in clinical practice: A retrospective cohort study. Kidney Medicine 5(3), 100600. 10.1016/j.xkme.2023.100600. PMC9984886 36879723

[102] Lees JS, Rutherford E, Stevens KI, Chen DC, Scherzer R, Estrella MM, Sullivan MK, Ebert N, Mark PB and Shlipak MG (2022) Assessment of cystatin C level for risk stratification in adults with chronic kidney disease. JAMA Network Open 5(10), e2238300. 10.1001/jamanetworkopen.2022.38300. PMC9597396 36282503

[103] Martin-Cleary C, Sanz AB, Avello A, Sanchez-Niño MD and Ortiz A (2023) NephroCheck at 10: Addressing unmet needs in AKI diagnosis and risk stratification. Clinical Kidney Journal 16(9), 1359–1366. 10.1093/ckj/sfad146. PMC10468756 37664563

[104] Castillo-Rodriguez E, Fernandez-Prado R, Martin-Cleary C, Pizarro-Sánchez MS, Sanchez-Niño MD, Sanz AB, Fernandez-Fernandez B and Ortiz A (2017) Kidney injury marker 1 and neutrophil gelatinase-associated lipocalin in chronic kidney disease. Nephron 136(4), 263–267. 10.1159/000447649. 27771693

[105] Romejko K, Markowska M and Niemczyk S (2023) The review of current knowledge on neutrophil gelatinase-associated lipocalin (NGAL). International Journal of Molecular Sciences 24(13). 10.3390/ijms241310470. PMC10341718 37445650

[106] Schulz CA, Engström G, Nilsson J, Almgren P, Petkovic M, Christensson A, Nilsson PM, Melander O and Orho-Melander M (2020) Plasma kidney injury molecule-1 (p-KIM-1) levels and deterioration of kidney function over 16 years. Nephrology, Dialysis, Transplantation: Official Publication of the European Dialysis and Transplant Association - European Renal Association 35(2), 265–273. 10.1093/ndt/gfy382. PMC7049260 30629206

[107] Pontillo C and Mischak H (2017) Urinary peptide-based classifier CKD273: Towards clinical application in chronic kidney disease. Clinical Kidney Journal 10(2), 192–201. 10.1093/ckj/sfx002. PMC5499684 28694965

[108] Good DM, Zürbig P, Argilés A, Bauer HW, Behrens G, Coon JJ, Dakna M, Decramer S, Delles C, Dominiczak AF, et al. (2010) Naturally occurring human urinary peptides for use in diagnosis of chronic kidney disease. Molecular & Cellular Proteomics: MCP 9(11), 2424–2437. 10.1074/mcp.M110.001917. PMC2984241 20616184

[109] Zürbig P, Mischak H, Menne J and Haller H (2019) CKD273 enables efficient prediction of diabetic nephropathy in Nonalbuminuric patients. Diabetes Care 42(1), e4–e5. 10.2337/dc18-1322. 30455331

[110] Tofte N, Lindhardt M, Adamova K, Bakker SJL, Beige J, Beulens JWJ, Birkenfeld AL, Currie G, Delles C, Dimos I, et al. (2020) Early detection of diabetic kidney disease by urinary proteomics and subsequent intervention with spironolactone to delay progression (PRIORITY): A prospective observational study and embedded randomised placebo-controlled trial. The Lancet Diabetes & Endocrinology 8(4), 301–312. 10.1016/S2213-8587(20)30026-7. 32135136

[111] Zhang J, Fuhrer T, Ye H, Kwan B, Montemayor D, Tumova J, Darshi M, Afshinnia F, Scialla JJ, Anderson A, Porter AC, Taliercio JJ, Rincon-Choles H, Rao P, Xie D, Feldman H, Sauer U, Sharma K and Natarajan L (2022) High-throughput metabolomics and diabetic kidney disease progression: Evidence from the chronic renal insufficiency (CRIC) study. American Journal of Nephrology 53(2–3), 215–225. 10.1159/000521940. PMC9116599 35196658

[112] Sharma K, Zhang G, Hansen J, Bjornstad P, Lee HJ, Menon R, Hejazi L, Liu JJ, Franzone A, Looker HC, Choi BY, Fernandez R, Venkatachalam MA, Kugathasan L, Sridhar VS, Natarajan L, Zhang J, Sharma VS, Kwan B, Waikar SS, Himmelfarb J, Tuttle KR, Kestenbaum B, Fuhrer T, Feldman HI, de Boer IH, Tucci FC, Sedor J, Heerspink HL, Schaub J, Otto EA, Hodgin JB, Kretzler M, Anderton CR, Alexandrov T, Cherney D, Lim SC, Nelson RG, Gelfond J, Iyengar R **and** Kidney Precision Medicine Project (2023) Endogenous adenine mediates kidney injury in diabetic models and predicts diabetic kidney disease in patients. The Journal of Clinical Investigation 133(20). 10.1172/JCI170341. PMC10575723 37616058

[113] Liu GY and Sabatini DM (2020) mTOR at the nexus of nutrition, growth, ageing and disease. Nature Reviews Molecular Cell Biology 21(4), 183–203. 10.1038/s41580-019-0199-y. PMC7102936 31937935

[114] Critselis E and Lambers Heerspink H (2016) Utility of the CKD273 peptide classifier in predicting chronic kidney disease *progression*. European Renal Association 31(2), 249–254. 10.1093/ndt/gfv062. 25791724

[115] Karger AB and Shlipak MG (2025) Glomerular filtration rate (GFR) estimation with cystatin C-past, present, and future. Clinical Chemistry 71(7), 743–751. 10.1093/clinchem/hvae226. 39902788

[116] Fu X, Shu Y and Zhang Y (2026) The potential of neutrophil gelatinase-associated lipocalin in management of acute kidney injury and peritoneal dialysis-related peritonitis: A narrative review. Frontiers in Nephrology 5, 1749827. 10.3389/fneph.2025.1749827. PMC12886388 41675263

[117] Su Y, Yang X, Cheng WW, Shang XM, Wang HL and Shen HC (2025) Kidney injury molecule 1 in the early detection of acute kidney injury-a systematic review and meta-analysis. Frontiers in Medicine 12, 1574945. 10.3389/fmed.2025.1574945. PMC12575230 41179873

[118] Zhang Z, Liu C, Han Y, Li D, Huang Y, Shi K, Xia S, Wei J, Liu H, Sun L, Zhu X and Yuan S (2025) The predictive value of suPAR for glomerular segmental sclerosis lesions in renal pathology. Renal Failure 47(1), 2498628. 10.1080/0886022X.2025.2498628. PMC12082739 40364434

[119] Reiser J, Hayek SS and Sever S (2026) The role of suPAR and related proteins in kidney, heart diseases, and diabetes. The Journal of Clinical Investigation 136(1). 10.1172/JCI197141. PMC12721894 41480757

[120] Kovesdy CP and Quarles LD (2013) Fibroblast growth factor-23: What we know, what we don’t know, and what we need to know. Nephrology, Dialysis, Transplantation: Official Publication of the European Dialysis and Transplant Association - European Renal Association 28(9), 2228–2236. 10.1093/ndt/gft065. PMC3769978 23625971

[121] Keller F, Schunk S, Denicolò S, Sharifli S, Thöni S, Eder S, Leierer J, Heerspink HJL, Mark PB, Rosivall L, Wiecek A, Mayer G and Fliser D (2026) Dickkopf-3 (DKK3) and the progression of diabetic kidney disease in primary health care. Diabetes 75(1), 144–153. 10.2337/db25-0235. PMC12716617 41071948

[122] Nanamatsu A, de Araújo L, LaFavers KA and el-Achkar TM (2024) Advances in uromodulin biology and potential clinical applications. Nature Reviews Nephrology 20(12), 806–821. 10.1038/s41581-024-00881-7. PMC11568936 39160319

[123] Wang W, Shen Q and Zhou X (2023) The predictive value of [TIMP-2]*[IGFBP7] in adverse outcomes for acute kidney injury: A systematic review and meta-analysis. Renal Failure 45(2), 2253933. 10.1080/0886022X.2023.2253933. PMC10512823 37724518

[124] Garimella PS, Lee AK, Ambrosius WT, Bhatt U, Cheung AK, Chonchol M, Craven T, Hawfield AT, Jotwani V, Killeen A, Punzi H, Sarnak MJ, Wall BM, Ix JH and Shlipak MG (2019) Markers of kidney tubule function and risk of cardiovascular disease events and mortality in the SPRINT trial. European Heart Journal 40(42), 3486–3493. 10.1093/eurheartj/ehz392. PMC6837159 31257404

[125] Mitsides N, Mitra V, Saha A, Harris S, Kalra PA and Mitra S (2023) Urinary liver-type fatty acid binding protein, a biomarker for disease progression, dialysis and overall mortality in chronic kidney disease. Journal of Personalized Medicine 13(10). 10.3390/jpm13101481. PMC10608048 37888092

[126] Satta E, Strollo F, Borgia L, Guarino G, Romano C, Masarone M, Marfella R and Gentile S (2025) Urinary L-FABP: A novel biomarker for evaluating diabetic nephropathy onset and progression. A narrative review. Diabetes Therapy: Research, Treatment and Education of Diabetes and Related Disorders 16(6), 1107–1124. 10.1007/s13300-025-01731-w. PMC12085547 40178792

[127] Ríos-Silva M, Huerta M, Mendoza-Cano O, Murillo-Zamora E, Cárdenas Y, Bricio-Barrios JA, Diaz Y, Ibarra I and Trujillo X (2023) Urinary epidermal growth factor in kidney disease: A systematic review. Nefrología 43(4), 413–426. 10.1016/j.nefro.2022.10.003.

[128] LeBleu VS, Teng Y, O’Connell JT, Charytan D, Müller GA, Müller CA, Sugimoto H and Kalluri R (2013) Identification of human epididymis protein-4 as a fibroblast-derived mediator of fibrosis. Nature Medicine 19(2), 227–231. 10.1038/nm.2989. PMC4457508 23353556

[129] Coca SG, Stapleton S, Edwards KL, Au ECK, Fleming F and Nadkarni GN (2024) Advancing KidneyIntelX precision medicine platform in CKD. Journal of the American Society of Nephrology 35(10S). 10.1681/ASN.2024kvck8cs8.

[130] Huang X, Yu N, Yu H and Yang S (2026) Association of growth differentiation factor-15 with renal disease outcomes and all-cause death in type 1 and type 2 diabetic patients: A meta-analysis. BMC Nephrology 27(1), 8. 10.1186/s12882-025-04624-z. PMC12763988 41291502

[131] Oshita T, Watanabe S, Toyohara T, Kujirai R, Kikuchi K, Suzuki T, Suzuki C, Matsumoto Y, Wada J, Tomioka Y, Tanaka T and Abe T (2023) Urinary growth differentiation factor 15 predicts renal function decline in diabetic kidney disease. Scientific Reports 13(1), 12508. 10.1038/s41598-023-39657-7. PMC10397309 37532799

[132] Ma R, Ouyang H, Chen C, Xu X, Tian J, Zhong R, Zha Y, Teng S, Li G, Liu H, Yang X and Hou FF (2025) Urinary cytokeratin 20 predicts severe acute kidney injury and major adverse kidney events in adults undergoing cardiac surgery. American Journal of Nephrology 56(6), 702–712. 10.1159/000546159. 40319884

[133] Ouyang H, Ma R, Yang X, Chen C, Xu X, Tian J, Liu J, Zha Y, Liu H, Yang T and Hou FF (2025) Urinary cytokeratin 20 as a biomarker for AKI-CKD transition among patients with acute decompensated heart failure and acute kidney injury. Journal of the American Society of Nephrology 36(3), 451–462. 10.1681/ASN.0000000518. PMC11888961 39466275

[134] Ma L, Choi YA, Li D, Saleem MA and Freedman BI (2026) Serum AKR1A1 levels predict eGFR decline rate in black Americans with type 2 diabetes. Kidney360 7(1), 131–138. 10.34067/KID.0000000966. PMC12889911 41051883

[135] Luo AJ, Chang FC and Lin SL (2024) Exploring Angiopoietin-2: Clinical insights and experimental perspectives in kidney diseases. Kidney International Reports 9(12), 3375–3385. 10.1016/j.ekir.2024.09.001. PMC11652073 39698365

[136] Sun P, Weng Q, Zhou J, Chen Q, Zhang R, and Nie J (2025) Identification of core genes in acute kidney injury: Evidence from multi-omics human transcriptomic data and in vivo models. Translational Andrology and Urology 14(5), 1327–1347. 10.21037/tau-2024-677. PMC12170083 40529024

[137] He Y, Zhang W, Li Z, Liu C, and Peng Y (2025) Hmox1 expression indicates distinct injury phenotypes of proximal tubule cells in sepsis-associated acute kidney injury. Scientific Reports 15(1), 37372, 10.1038/s41598-025-05764-w. PMC12559336 41145479

[138] Jiang K, Mai S, Li J, Zhou H, Chen Y, Zou L and Yu H (2025) Exploring the molecular mechanisms of lactylation-related biological functions and immune regulation in sepsis-associated acute kidney injury. Clinical and Experimental Medicine 25(1), 200. 10.1007/s10238-025-01745-5. PMC12162736 40504273

[139] Liu X, Li Z, Mao L, Lu Y, Cheng L, Xin X, Jiang L, Wang Z, Sun P, Xu L, Zheng X, Xiang C, Chen Y, Wang S, Liu G and Yang L (2025) Integration of single-cell RNA sequencing and spatial transcriptomics reveals neutrophil diversity and spatial heterogeneity in acute kidney injury. International Journal of Biological Macromolecules 331(Pt 2), 148478. 10.1016/j.ijbiomac.2025.148478. 41135905

[140] Zhang YL, Tang TT, Wang B, Wen Y, Feng Y, Yin Q, Jiang W, Zhang Y, Li ZL, Wu M, Wu QL, Song J, Crowley SD, Lan HY, Lv LL and Liu BC (2024) Identification of a novel ECM Remodeling macrophage subset in AKI to CKD transition by integrative spatial and single-cell analysis. Advanced Science (Weinheim, Baden-Württemberg, Germany) 11(38), e2309752. 10.1002/advs.202309752. PMC11481374 39119903

[141] Gerhardt LMS, Koppitch K, van Gestel J, Guo J, Cho S, Wu H, Kirita Y, Humphreys BD and McMahon AP (2023) Lineage tracing and single-nucleus multiomics reveal novel features of adaptive and maladaptive repair after acute kidney injury. Journal of the American Society of Nephrology 34(4), 554–571. 10.1681/ASN.0000000000000057. PMC10103206 36735940

[142] Zheng X, Guo X, Chen Y, Zhuang K, Gong N, Fu Y, Liang Y, Xu Y, Wang S, Wang W, Chen X and Cai G (2025) Integration of DNA methylome and transcriptome analysis to identify novel epigenetic targets in the acute kidney injury-chronic kidney disease transition. Biomolecules 15(4). 10.3390/biom15040498. PMC12024732 40305204

[143] Lake BB, Melo Ferreira R, Hansen J, Menon R, Basta J, Thiessen Philbrook H, Reinert S, Fallegger R, Lagwankar AK, Chen X, et al. (2025) Cellular and spatial drivers of unresolved injury and functional decline in the human kidney. bioRxiv: The Preprint Server for Biology.

[144] Strauß C, Booke H, Forni L and Zarbock A (2024) Biomarkers of acute kidney injury: From discovery to the future of clinical practice. Journal of Clinical Anesthesia 95, 111458. 10.1016/j.jclinane.2024.111458. 38581927

[145] Wu Q, Qiu X and Chen H (2026) Advancements in early biomarkers of acute kidney injury: From traditional indicators to a paradigm shift in lactate metabolism. Annals of Medicine 58(1), 2612383. 10.1080/07853890.2025.2612383. PMC12805857 41530927

[146] Shi K, Jiang W, Song L, Li X, Zhang C, Li L, Feng Y, Yang J, Wang T, Wang H, Zhou L, Yu J and Zheng R (2025) Persistent acute kidney injury biomarkers: A systematic review and meta-analysis. Clinica Chimica Acta; International Journal of Clinical Chemistry 564, 119907. 10.1016/j.cca.2024.119907. 39127297

[147] Demirjian S, Chawla L, Davison D, Forni LG, Heung M, Hoste EAJ, Koyner J, Kampf JP, Kwan T, McPherson P and Kellum JA (2024) CCL14 predicts oliguria and dialysis requirement in patients with moderate to severe acute kidney injury. Blood Purification 53(7), 548–556. 10.1159/000538898. PMC11239143 38636476

[148] Wang X, Huang P, Luo Y, Xin Y, Li Y, Shen L, Liu Y, Zhou Y, Zhang Y, Zhang Q, Wang D, Luan F, Zhang W, Yuan M, Liu Y, Liu F, Zhang N, Wu J, Wu T, Wang X, Bai Y, Zhao M, Wang C and Yu K (2025) Urinary 3-methylhistidine as a potential biomarker for sepsis-associated acute kidney injury: Multidimensional metabolomics analysis in mice and human. Annals of Intensive Care 15(1), 125. 10.1186/s13613-025-01550-z. PMC12380662 40858915

[149] Li N, Tang TT, Gu M, Fu YQ, Qian WW, Ma NN, Shen AR, Zhu T, Huo RB, Zhang T, Xie JF, Zhang L, Liu BC and Lv LL (2025) Single urinary extracellular vesicle proteomics identifies complement receptor CD35 as a biomarker for sepsis-associated acute kidney injury. Nature Communications 16(1), 6960. 10.1038/s41467-025-62229-4. PMC12307914 40730843

[150] Reznichenko A, Nair V, Eddy S, Fermin D, Tomilo M, Slidel T, Ju W, Henry I, Badal SS, Wesley JD, Liles JT, Moosmang S, Williams JM, Quinn CM, Bitzer M, Hodgin JB, Barisoni L, Karihaloo A, Breyer MD, Duffin KL, Patel UD, Magnone MC, Bhat R and Kretzler M (2024) Unbiased kidney-centric molecular categorization of chronic kidney disease as a step towards precision medicine. Kidney International 105(6), 1263–1278. 10.1016/j.kint.2024.01.012. PMC11751912 38286178

[151] Odenkirk MT, Reif DM and Baker ES (2021) Multiomic big data analysis challenges: Increasing confidence in the interpretation of artificial intelligence assessments. Analytical Chemistry 93(22), 7763–7773. 10.1021/acs.analchem.0c04850. PMC8465926 34029068

[152] El-Achkar TM, Eadon MT, Menon R, Lake BB, Sigdel TK, Alexandrov T, Parikh S, Zhang G, Dobi D, Dunn KW, et al. (2021) A multimodal and integrated approach to interrogate human kidney biopsies with rigor and reproducibility: Guidelines from the kidney precision medicine project. Physiological Genomics 53(1), 1–11. 10.1152/physiolgenomics.00104.2020. PMC7847045 33197228

[153] Tarazona S, Arzalluz-Luque A and Conesa A (2021) Undisclosed, unmet and neglected challenges in multi-omics studies. Nature Computational Science 1(6), 395–402. 10.1038/s43588-021-00086-z. 38217236

[154] Pirmoradi S, Teshnehlab M, Zarghami N and Sharifi A (2021) A self-organizing deep neuro-fuzzy system approach for classification of kidney cancer subtypes using miRNA genomics data. Computer Methods and Programs in Biomedicine 206, 106132. 10.1016/j.cmpb.2021.106132. 34010800

[155] Imoto Y (2025) Comprehensive noise reduction in single-cell data with the RECODE platform. Cell Reports Methods 5(10), 101178. 10.1016/j.crmeth.2025.101178. PMC12570323 40967209

[156] Zhou K, Tang Z and Hu J (2026) Biomarker-driven risk stratification and early intervention in acute kidney injury: A comprehensive review from early warning to clinical response. Frontiers in Medicine 13, 1796756. 10.3389/fmed.2026.1796756. PMC13021607 41907247

[157] KDIGO (2024) KDIGO 2024 clinical practice guideline for the evaluation and Management of Chronic Kidney Disease. Kidney International 105(4), S117–s314. 10.1016/j.kint.2023.10.018. 38490803

[158] Yeo SC, Wang H, Ang YG, Lim CK and Ooi XY (2024) Cost-effectiveness of screening for chronic kidney disease in the general adult population: A systematic review. Clinical Kidney Journal 17(1), sfad137. 10.1093/ckj/sfad137. PMC10765095 38186904

[159] Xie W, Zhao D, Franz H, Schmitt A, Walz G and Yakulov TA (2026) Multi-omics integration identifies TNFRSF1A as a causal mediator of immune microenvironment reprogramming in diabetic kidney disease. International Journal of Molecular Sciences 27(1), 27. 10.3390/ijms27010279. PMC12785335 41516155

[160] Yiu T, Chen B, Wang H, Feng G, Fu Q and Hu H (2025) Transformative advances in single-cell omics: A comprehensive review of foundation models, multimodal integration and computational ecosystems. Journal of Translational Medicine 23(1), 1176. 10.1186/s12967-025-07091-0. PMC12560279 41146276

[161] Sims JK, Manteiga S and Lee K (2013) Towards high resolution analysis of metabolic flux in cells and tissues. Current Opinion in Biotechnology 24(5), 933–939. 10.1016/j.copbio.2013.07.001. PMC3783555 23906926

[162] Hammoud S, Kern J, Mukherjee S, Lutkewitte AJ, Singh P, Newberry K, Finck BN and Gewin LS (2025) Assays to enhance metabolic phenotyping in the kidney. American Journal of Physiology 328(4), F563–f577. 10.1152/ajprenal.00232.2024. PMC12145867 39819047

[163] Hahn O, Chrysopoulou M, Jaegers J, Laustsen C and Rinschen MM (2024) Metabolic alterations in organs and biofluids following unilateral ischemia-reperfusion kidney injury in pigs. Journal of the American Society of Nephrology 35(10S). 10.1681/ASN.2024dgbs189a.

[164] Qiao Y, Zhou H, Liu Y, Chen R, Zhang X, Nie S, Hou FF, Zhao Y, Xu X and Zhao L (2024) A multi-modal fusion model with enhanced feature representation for chronic kidney disease progression prediction. Briefings in Bioinformatics 26(1). 10.1093/bib/bbaf003. PMC11801269 39913621

[165] Tangri N, Cheungpasitporn W, Crittenden SD, Fornoni A, Peralta CA, Singh K, Usvyat LA, Waterman AD **and** on behalf of the American Society of Nephrology (ASN) Artificial Intelligence (AI) Workgroup (2026) Responsible use of artificial intelligence to improve kidney care. Journal of the American Society of Nephrology 37(4), 881–890. 10.1681/ASN.0000000929. PMC13065138 41201255

[166] Liu M, Li Y, Sun L, Sun M, Hu X, Li Q, Yu M, Wang C, Ren X and Ma J (2024) Integrating multi-organ imaging-derived phenotypes and genomic information for predicting the occurrence of common diseases. Bioengineering (Basel) 11(9). 10.3390/bioengineering11090872. PMC11428582 39329614

[167] de Boer IH, Alpers CE, Azeloglu EU, Balis UGJ, Barasch JM, Barisoni L, Blank KN, Bomback AS, Brown K, Dagher PC et al. (2021). Rationale and design of the kidney precision medicine project. Kidney International 2021, 99(3):498–510. 10.1016/j.kint.2020.08.039. PMC8330551 33637194

[168] Anandakrishnan N, Yi Z, Sun Z, Liu T, Haydak J, Eddy S, Jayaraman P, DeFronzo S, Saha A, Sun Q, et al. (2025) Liquid biopsy-multiomics link adhesion pathway dysregulation to kidney injury severity. Kidney International Reports 10(10), 3592–3610. 10.1016/j.ekir.2025.07.021. PMC12545944 41141506

